# Persistent ER stress causes GPI anchor deficit to convert a GPI-anchored prion protein into pro-PrP *via* the ATF6–miR449c-5p–PIGV axis

**DOI:** 10.1016/j.jbc.2023.104982

**Published:** 2023-06-28

**Authors:** JingFeng Li, SaSa Li, ShuPei Yu, Jie Yang, JingRu Ke, Huan Li, Heng Chen, MingJian Lu, Man-Sun Sy, ZhenXing Gao, Chaoyang Li

**Affiliations:** 1 Wuhan Institute of Virology, Chinese Academy of Sciences, State Key Laboratory of Virology, Wuhan, China; 2 University of Chinese Academy of Sciences, Beijing, China; 3 Affiliated Cancer Hospital and Institute of Guangzhou Medical University, State Key Laboratory of Respiratory Disease, Guangzhou, China; 4 Department of Dermatology, Tongji Hospital, Tongji Medical College, Huazhong University of Science and Technology, Wuhan, China; 5 Department of Interventional Radiology, Affiliated Cancer Hospital and Institute of Guangzhou Medical University, Guangzhou, China; 6 Department of Pathology, School of Medicine, Case Western Reserve University, Cleveland, Ohio, USA

**Keywords:** prion protein, pro-protein, ER stress, unfolded protein response, miRNA, GPI-anchored proteins, PDAC

## Abstract

Endoplasmic reticulum (ER) stress and unfolded protein response are cells’ survival strategies to thwart disruption of proteostasis. Tumor cells are continuously being challenged by ER stress. The prion protein, PrP, normally a glycosylphosphatidylinositol (GPI)-anchored protein exists as a pro-PrP retaining its GPI-peptide signal sequence in human pancreatic ductal cell adenocarcinoma (PDAC). Higher abundance of pro-PrP indicates poorer prognosis in PDAC patients. The reason why PDAC cells express pro-PrP is unknown. Here, we report that persistent ER stress causes conversion of GPI-anchored PrP to pro-PrP via a conserved ATF6–miRNA449c-5p–PIGV axis. Mouse neurons and AsPC-1, a PDAC cell line, express GPI-anchored PrP. However, continuous culture of these cells with the ER stress inducers thapsigargin or brefeldin A results in the conversion of a GPI-anchored PrP to pro-PrP. Such a conversion is reversible; removal of the inducers allows the cells to re-express a GPI-anchored PrP. Mechanistically, persistent ER stress increases the abundance of an active ATF6, which increases the level of miRNA449c-5p (miR449c-5p). By binding the mRNA of PIGV at its 3′-UTRs, miR449c-5p suppresses the level of PIGV, a mannosyltransferase pivotal in the synthesis of the GPI anchor. Reduction of PIGV leads to disruption of the GPI anchor assembly, causing pro-PrP accumulation and enhancing cancer cell migration and invasion. The importance of ATF6–miR449c-5p–PIGV axis is recapitulated in PDAC biopsies as the higher levels of ATF6 and miR449c-5p and lower levels of PIGV are markers of poorer outcome for patients with PDAC. Drugs targeting this axis may prevent PDAC progression.

The normal cellular prion protein (PrP) is a highly conserved and widely expressed glycosylphosphatidylinositol (GPI)-anchored cell surface glycoprotein (GPI-AP) (1). Most noticeably, the expression of PrP is pivotal for the development of an infectious neurodegeneration disease commonly referred to as prion disease (2, 3, 4, 5). However, the normal physiologic functions of PrP remain elusive (2). PrP is first synthesized as a pre-pro-PrP. At the N terminus, there is a signal peptide, and at the C-terminus, there is a GPI-peptide signal sequence (GPI-PSS). Removal of the signal peptide generates pro-PrP, which still retains the GPI-PSS. Cleavage of the GPI-PSS at the ω site (Gly 229 in human PrP) allows the attachment of an already assembled GPI anchor completing the GPI anchor modification process and transmits to the cell surface (6). All these processes occur in the endoplasmic reticulum (ER) (7).

Previously, we reported that in the normal human pancreas, only islet cells express detectable PrP; neither ductal cells nor acinar cells have detectable PrP (8, 9). Although human pancreatic ductal adenocarcinoma (PDAC) cells are thought to derive from ductal cells, about 45% of the human PDAC biopsies express high levels of PrP (8). Furthermore, all PDAC cell lines (N = 7) studied also express PrP. However, in these cell lines as well as in PDAC biopsies, the PrP expressed is a pro-PrP instead of a GPI-anchored PrP (8). Subsequently, our findings in human PDAC were recapitulated in human melanomas (10, 11). While normal human melanocyte does not express detectable PrP, about 50% of human melanomas biopsies and all eight human melanoma cell lines have high levels of pro-PrP (10, 11). In both cases, the pro-PrP is present on the cell surface using the highly hydrophobic GPI-PSS as a transmembrane domain. Furthermore, higher expression of PrP is a marker of poorer prognosis in patients with PDAC as well as in patients with melanomas (8, 11). However, pro-PrP in PDAC and melanoma engages distinct pathways to alter cancer cell biology. In PDAC cell lines BxPC3 and Panc02.03, the PrP-GPI-PSS interacts with filament A (FLNa), a cytolinker protein critical in cytoskeleton organization (8). In melanoma, the PrP-GPI-PSS interacts with casitas B-lineage lymphoma (c-Cbl), an E3 ubiquitin ligase important in tyrosine kinase receptor signaling (11). Despite these differences, the consequences of these interactions are similar, rendering the tumor cells more aggressive.

Since the precursors of PDAC and melanoma are mostly PrP negative, two events must occur during the transformation processes, the increase of PrP expression and the failure to remove the GPI-PSS. More than 150 GPI-APs have been confirmed in the human genome (12). The process of GPI anchor modification is complex and involved about 30 genes and is not completely understood (13). Intriguingly, in comparison to nine other human GPI-PSSs from other GPI-APs, the GPI-PSS of PrP is the least efficient in accepting the assembled GPI anchor in an *in vitro* GPI anchor modification assay (14). Thus, GPI modification of PrP may be more predisposed to disruption due to a slight deficit in the GPI anchor modification machinery than other GPI-APs. Furthermore, unlike most of the other GPI-APs which enter ER independent of SEC62 and SEC63, PrP’s entry is mediated by SEC62 and SEC63 (15). In addition, some of the enzymes required for GPI modifications may be protein context–dependent (15). Thus, different GPI-APs may follow distinct biosynthetic as well as trafficking pathways. Most cancer cells show signs of ER stress as a result of internal and environmental constraints (16). Since the GPI-anchor modification process occurs mostly in the ER, a disruption in the ER proteostasis, such as ER stress, may contribute to the accumulation of pro-PrP in tumor cells.

Till now, AsPC-1 is the only PDAC cell line out of eight, which expresses a normal GPI-anchored PrP (17). To mimic persistent ER stress in tumors, we treated AsPC-1 cells with 30 nM thapsigargin (Tg) or 70 nM brefeldin A (BFA) for various lengths of time. We find that culturing the cells with Tg and BFA for 14 days indeed induces the accumulation of pro-PrP without increasing the expression of total PrP. Consistent with earlier results, expression of pro-PrP leads to higher motility and invasiveness of the cancer cells *in vitro*. However, after the removal of Tg or BFA and continuing culture the cells in the normal medium, the AsPC-1 cells re-express GPI-anchored PrP. Mechanistically, we show that ER stress increases the level of cleaved activating transcription factor 6 (c-ATF6), which then augments the abundance of miR 449c-5p. miR449c-5p binds to the 3′UTR of phosphatidylinositol-glycan biosynthesis class V protein (PIGV) mRNA mitigating its mRNA and thus reducing the level of PIGV, a mannosyltransferase pivotal in the synthesis of the GPI anchor core. The same phenomenon is detected in a mouse neural cell line (MNC) when persistent ER stress is induced. Finally, we show that our finding in cell models has clinical relevance. Higher abundance of miR449c-5p and activating transcription factor (ATF)6 and lower abundance of PIGV are markers of poorer prognosis in patients with PDAC.

## Results

### Persistent Tg and BFA treatment induces pro-PrP accumulation increasing cancer cell migration and invasion *in vitro*

**Figure 1:**
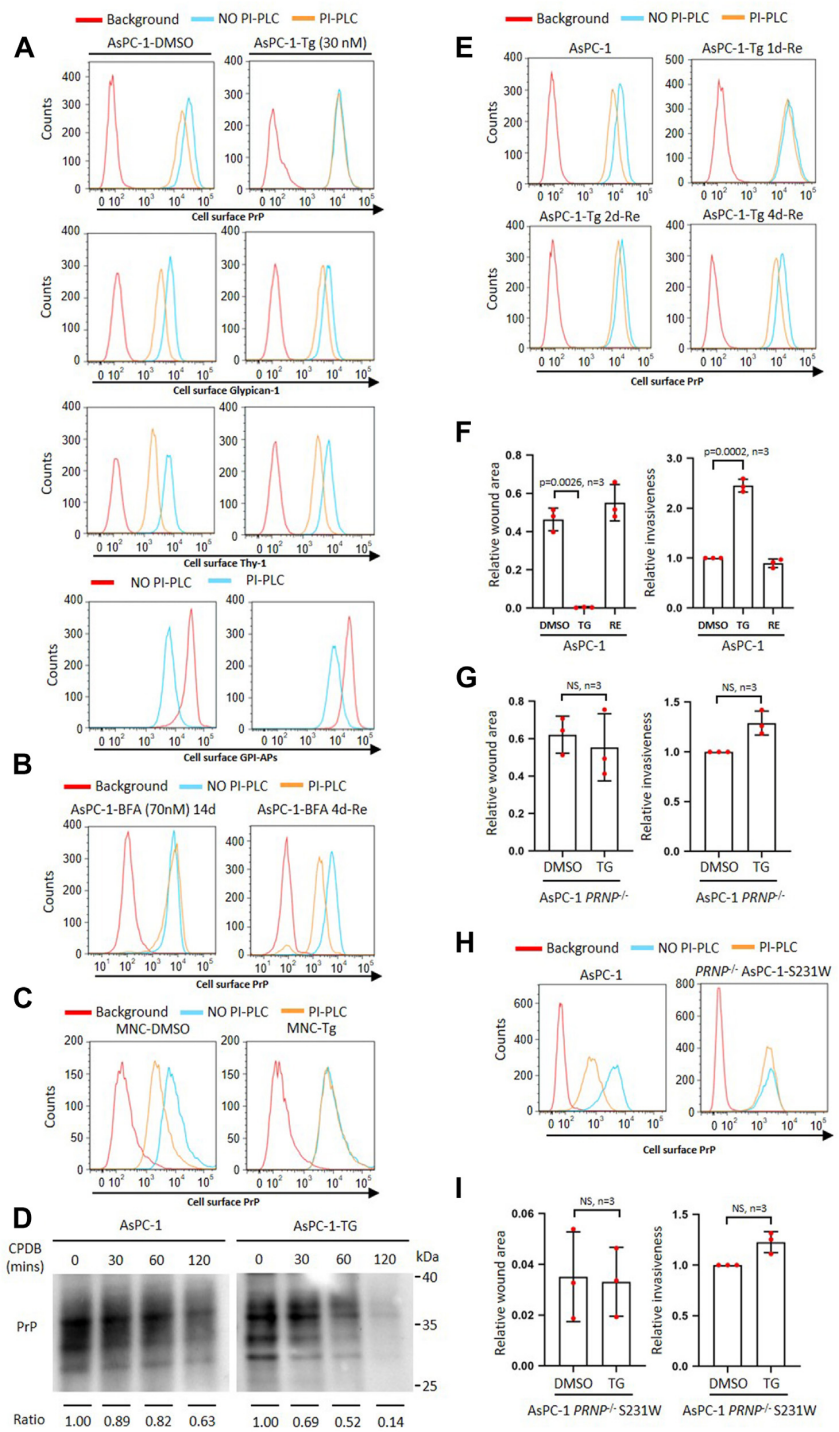
**Persistent Tg and BFA treatment induces pro-PrP accumulation increasing AsPC-1 cancer cell migration and invasion *in vitro*.***A*, persistent Tg treatment (AsPC-1-Tg (30 nM)) specifically causes cell surface PrP to be resistant to PI-PLC treatment. Row 1: PrP on the surface of Tg but not DMSO-treated AsPC-1 cells showed resistance to PI-PLC treatment. Rows 2&3&4: Glypican-1, Thy-1, and GPI-APs on the surface of Tg and DMSO-treated AsPC-1 cells were still sensitive to PI-PLC treatment. *B*, persistent BFA treatment (AsPC-1-BFA (70 nM) 14d) induces pro-PrP accumulation, while 4 days post removal of BFA (AsPC-1-BFA 4d-RE) causes cell surface PrP to be sensitive to PI-PLC treatment. *C*, persistent Tg treatment (MNC-Tg) induces pro-PrP accumulation in a mouse neuronal cell line (MNC). *D*, persistent Tg treatment causes PrP purified from AsPC-1 to be sensitive to CPDB treatment. Ratio: PrP levels quantified by Image J at indicated time point to PrP level at time 0. *E*, removal of Tg from culture medium (AsPC-1-Tg-RE) resensitizes cell surface PrP to be sensitive to PI-PLC treatment. *F*, persistent Tg treatment causes AsPC-1 cancer cell migrating significantly faster and more invasive, while removal of Tg decreases the migratory and invasive capability. *G*, persistent Tg treatment does not increase the mobility and invasion of AsPC-1 *PRNP*^−/−^ cancer cell. *H*, S231W-mutated PrP is resistant to PI-PLC treatment. *I*, persistent Tg treatment does not significantly increase motility and invasion of AsPC-1 cells expressing S231W-mutated PrP. Background: Cells not treated with PI-PLC but stained with the same concentration of IgG1 as the primary antibodies indicated. The data were expressed as mean ± SD and were analyzed by two-tailed double sample heteroscedasticity Student's *t* test. TG: Cells with Tg treatment for 14 days. RE: TG cells cultured for four without Tg treatment. TG and RE are defined the same in Figures 2, 3 and 5. CPDB, carboxypeptidase B; GPI, glycosylphosphatidylinositol; GPI-AP, GPI-anchored protein; NS, not significant; PrP, prion protein; PI-PLC, phosphoinositide-specific phospholipase C; Tg, thapsigargin.

Persistent ER stress which disturbs proteostasis inside ER has been observed in many types of cancers (16). ER stress has been shown to affect GPI-anchor synthesis (18); we posited that persistent ER stress may contribute to the accumulation of pro-PrP. To investigate such a possibility, we treated AsPC-1 cells with different concentrations of Tg and detected the effects on PrP expression. We initially found that treatment of AsPC-1 with Tg ranging from 30 to 500 nM for 24 h did not significantly enhance PrP levels (Fig. S1*A*). Since 30 nM Tg did not cause significant cytotoxicity as evidenced by the cell proliferation assays (Fig. S1*B*), we decided on this concentration to induce persistent ER stress in AsPC-1 cells. We treated AsPC-1 cells for various lengths of time; we found that, as treatment proceeded, cell surface PrP became less and less sensitive to phosphoinositide-specific phospholipase C (PI-PLC) treatment (Fig. S1*C*). Fourteen days post-Tg treatment (AsPC-1 cells maintained in Tg longer than 14 days were designated as AsPC-1-Tg or simply TG), the PrP on the AsPC-1-Tg cell surface became PI-PLC–resistant (Fig. 1*A*, row 1 and Fig. S1*D*) while the total PrP level was not impacted (Fig. S1*E*), an indication of a lack of a GPI anchor and a feature of pro-PrP. In contrast, the PrP on control, dimethyl sulfoxide (DMSO)-treated cells remained PI-PLC sensitive (Fig. 1*A*, top left panel, and Fig. S1*D*). We also determined whether the effect of ER stress only happens to PrP. Staining for glypican-1 and Thy-1, two normally GPI-APs, showed only marginal effects (Fig. 1*A*, rows 2 and 3, and Fig. S1*D*). Similar to glypican-1 and Thy-1, most of other GPI-APs on the cell surface remain sensitive to PI-PLC treatment as evidenced by FLAER staining, which reacts with GPI anchors (Fig. 1*A*, row 4, and Fig. S1*D*).

To assess if this phenomenon can be applied to other ER stress inducers, we treated AsPC-1 with low dose BFA (70 nM) for 14 days (AsPC-1 cells maintained in BFA longer than 14 days were designated as AsPC-1-BFA) and found that this treatment also resulted in PI-PLC–resistant PrP on the cell surface (Fig. 1*B*, left panel; Fig. S1*F*). To address whether similar changes occur in other cell types, we treated a PrP-overexpressed MNC (9) with Tg (MNC cells maintained in Tg longer than 14 days were designated as MNC-Tg). The results showed that Tg treatment also caused PrP on the cell surface to become PI-PLC resistant, while PrP on the cell surface of control-treated MNC was still sensitive to PI-PLC (Figs. 1*C* and S1*G*). Thus, persistent ER stress not only causes the accumulation of pro-PrP on the cell surface of a tumor cell line, it also has similar effects on a normal MNC.

Previously, it has been reported that persistent ER stress causes a decrease in the interaction between GPI-APs and post-GPI attachment to proteins factor 1 (PGAP1), an enzyme which catalyzes the inositol deacylation of GPI, resulting in the generation of inositol-acylated GPI-APs on the cell surface, which are resistant to PI-PLC treatment (18). To differentiate whether the PrP on the cell surface of AsPC-1-Tg/AsPC-1-BFA cells is inositol-acylated, we purified PrP from AsPC-1-Tg/AsPC-1-BFA cells and performed carboxypeptidase B (CPDB) treatment. Inositol-acylated GPI anchor is resistant to CPDB, while pro-PrP lacking the GPI anchor would be sensitive (19). We found that PrP from AsPC-1-Tg/AsPC-1-BFA cells was sensitive to CPDB, while PrP from AsPC-1 cells was resistant to CPDB (Figs. 1*D* and S1*H*, middle panel). Therefore, the PrP in AsPC-1-Tg cell is pro-PrP rather than the inositol-acylated, GPI-anchored PrP. Collectively, these observations support the conclusion that persistent ER stress leads to the accumulation of pro-PrP in AsPC-1 cells as well as in a normal MNC.

We then assessed whether the effects of persistent ER stress in pro-PrP accumulation is permanent or reversible. We cultured AsPC-1-Tg cells/AsPC-1-BFA cells in a medium without Tg or BFA (designated as AsPC-1-Tg/BFA-Re cells) for different lengths of time. We found that the longer the AsPC-1-Tg-Re cells were cultured, the more sensitive the cell surface PrP was to PI-PLC treatment (Figs. 1*E* and S1*I*); culturing cells without Tg or BFA for 4 days (designated as RE) appeared to be sufficient in rendering cell surface PrP to be PI-PLC–sensitive, again without altering the levels of PrP (Fig. 1*B*, right panel, Figs. 1*E*, and S1, *E* and *I*). Thus, the effect of persistent ER stress in pro-PrP accumulation is reversible when the stress signal is removed.

Expression of a pro-PrP confers a tumor cell motility and invasion advantages (8, 11, 17). We then compared the cell migration capabilities of AsPC-1, TG, and RE cells. We found that TG migrated significantly faster than AsPC-1 and RE cells (Figs. 1*F* and S1*J*). We also assessed the *in vitro* invasion capabilities of AsPC-1, TG, and RE cells. TG cells showed significantly higher invasive capability than AsPC-1 and RE cells (Figs. 1*F* and S1*J*). To investigate whether an enhanced mobility and invasiveness of TG cells is solely due to the presence of pro-PrP, we knocked out *PRNP* in AsPC-1 and treated the cells for 14 days (designated as AsPC-1-*PRNP* ^−/−^ and AsPC-1-*PRNP* ^−/−^-Tg, respectively) (Fig. S1*K*); we found that indeed AsPC-1 *PRNP*^−/−^ and AsPC-1-*PRNP* ^−/−^-Tg cells did not show significant differences in migration and invasion (Figs. 1*G* and S1*K*). Therefore, the expression of pro-PrP is pivotal for the enhanced migration and invasion of the AsPC-1-Tg cells.

There are more than 150 human GPI-anchored proteins (20). ER stress could have also resulted in the production of pro-proteins other than PrP. To investigate whether the differences in motility and invasiveness between AsPC-1 and TG cells were due to the conversion of GPI-anchored PrP to pro-PrP, we reintroduced an S231W mutant *PRNP* plasmid back into AsPC-1-*PRNP* ^−/−^ cells (Fig. S1*L*) (designated as AsPC-1-*PRNP* ^−/−^-S231W). Because of the mutation being on a ω+2 site, which is critical for the cleavage of the GPI-PSS (6, 21), this mutation renders the GPI-PSS to be resistant to transamidase, and the PrP expressed is a pro-PrP and thus resistant to PI-PLC but sensitive to CPDB (Figs. 1*H* and S1, *M* and *H*, right panel). Indeed, Tg treatment did not further increase the migration and invasion of AsPC-1-*PRNP*^−/−^-S231W cells (Figs. 1*I* and S1*L*). Collectively, these results imply that persistent ER stress–induced pro-PrP accumulation is vital in conferring migration and invasiveness advantages for the PDAC cells. Some other GPI-APs may be impacted during the ER stress; however, they appear to play a less critical role in the migration and invasion of AsPC-1 cells.

### Tg treatment reduces PIGV resulting in the accumulation of pro-PrP

**Figure 2:**
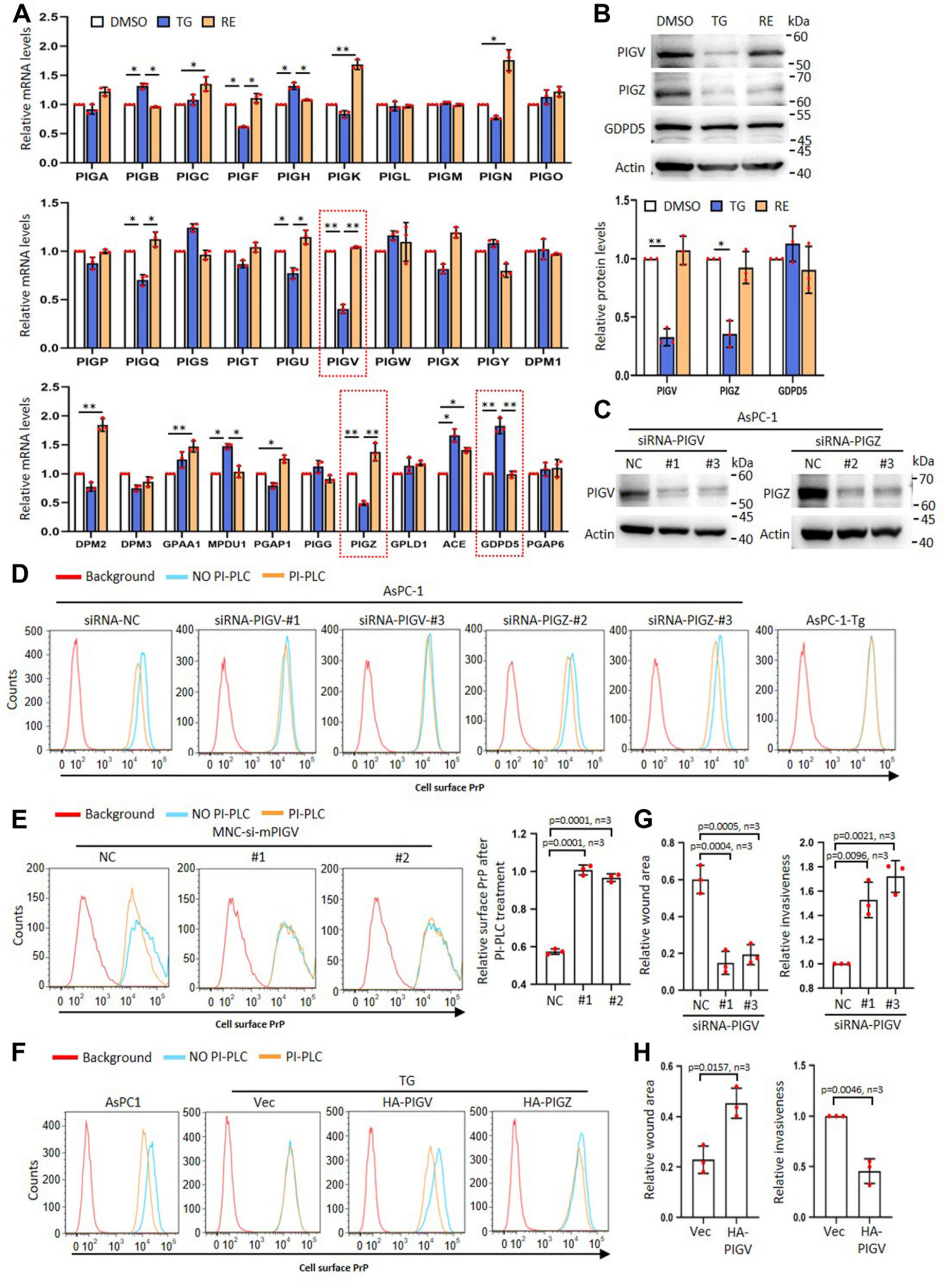
**Tg treatment reduces PIGV resulting in the accumulation of pro-PrP.***A*, QPCR analysis of GPI-anchor synthesis–related gene expression of DMSO, TG, and RE AsPC-1 cells. Genes with significant higher abundance of two-fold differences are *circles* in *red rectangle* with *dashed line*. *B*, immunoblotting analysis of PIGV, PIGZ, and GDPD5 of DMSO, TG, and RE AsPC-1 cells (*upper panel*). Statistical analysis of immunoblot results (*bottom panel*). *C*, immunoblotting analysis of siRNA-silenced AsPC-1 cells. *D*, flow cytometry analysis of cell surface PrP after siRNA silencing PIGV and PIGZ in AsPC-1 cells treated with or without PI-PLC. *E*, flow cytometry analysis of cell surface PrP after siRNA silencing PIGV in MNC with or without PI-PLC. Statistical analysis of flow cytometry results is shown. Relative surface PrP after PI-PLC treatment defined as: (geometry mean of PrP intensity after PI-PLC treatment - background geometry mean of PrP intensity)/(geometry mean of PrP intensity before PI-PLC treatment - background geometry mean of PrP intensity). *F*, flow cytometry analysis of cell surface PrP after expressing an HA-tagged PIGV or PIGZ in TG cells with or without PI-PLC. *G*, statistical analysis of motility and invasiveness assays after silencing PIGV in AsPC-1 cancer cells. *H*, statistical analysis of motility and invasiveness assays after expressing an HA-tagged PIGV in AsPC-1-Tg cells. The data were expressed as mean ± SD and were analyzed by two-tailed double sample heteroscedasticity Student's *t* test. ∗*p*< 0.05 and ∗∗*p*< 0.01. GDPD5, glycerophosphodiester phosphodiesterase domain-containing protein 5; NS, not significant; NC, non-targeting siRNA control; MNC, mouse neural cell line; PrP, prion protein; PIGV, phosphatidylinositol-glycan biosynthesis class V protein; PI-PLC, phosphoinositide-specific phospholipase C; PIGZ, , phosphatidylinositol-glycan biosynthesis class Z protein; Tg, thapsigargin; Vec, empty vector control.

The biosynthesis of GPI-APs is controlled by the balance of three major factors, amounts of GPI and precursor proteins, and GPI transamidase activity (22). Persistent ER stress has been reported to impact GPI-anchor synthesis (18). We thus investigated whether persistent ER stress affected the expression of the enzymes involved in the GPI anchor synthesis. Deep sequencing results showed that expression of these genes was affected by the presence of Tg (Fig. S2*A*). Quantitative polymerase chain reaction results revealed that among the 31 genes evaluated, the expression of 16 genes had significant changes and the expression patterns of 14 genes were reversed 4 days after Tg was removed from the culture medium (Fig. 2*A*). Among these 14 genes, only PIGV, phosphatidylinositol-glycan biosynthesis class Z protein (PIGZ), and glycerophosphodiester phosphodiesterase domain-containing protein 5 (GDPD5) consistently showed at least two-fold changes (Fig. 2*A*, in red square). We then performed immunoblotting analysis to detect the protein levels of these three genes. The results showed that the protein levels of PIGV and PIGZ were significantly decreased in TG cells and increased 4 days after the removal of Tg (Fig. 2*B*). In comparison, GDPD5 protein levels were not altered by a change in the culture condition (Fig. 2*B*).

To assess whether PIGZ or/and PIGV expression affected the accumulation of pro-PrP, we silenced PIGV or PIGZ, respectively, with siRNA (Figs 2*C* and S2*B*). We found that only silencing PIGV but not PIGZ caused cell surface PrP to become PI-PLC–resistant (Figs. 2*D* and S2*C*). We also silenced PIGV in MNC with siRNA (Fig. S2*D*). We found that decreasing mouse PIGV levels also significantly reduced cell surface PrP sensitivity to PI-PLC (Fig. 2*E*). To further confirm the effects of the expression of PIGV or PIGZ on PrP, we overexpressed an HA-tagged PIGV or PIGZ in TG cells (Fig. S2*E*) and detected the sensitivity of cell surface PrP to PI-PLC treatment. We found that overexpressing PIGV but not PIGZ rendered the PrP to become PI-PLC–sensitive (Figs. 2*F* and S2*F*). Collectively, these results strongly support the conclusion that Tg treatment decreased PIGV protein expression causing the accumulation of pro-PrP.

We then investigated whether alteration of PIGV protein levels might affect cancer cell migration and invasiveness *in vitro*. The results showed that silencing PIGV significantly enhanced the mobility and invasiveness of AsPC-1 cells (Figs. 2*G* and S2*G*), while overexpressing PIGV significantly impacted the motility and invasion of TG cells (Figs. 2*H* and S2*H*).

### Tg treatment increases ATF6 to reduce PIGV, resulting in the accumulation of pro-PrP

**Figure 3:**
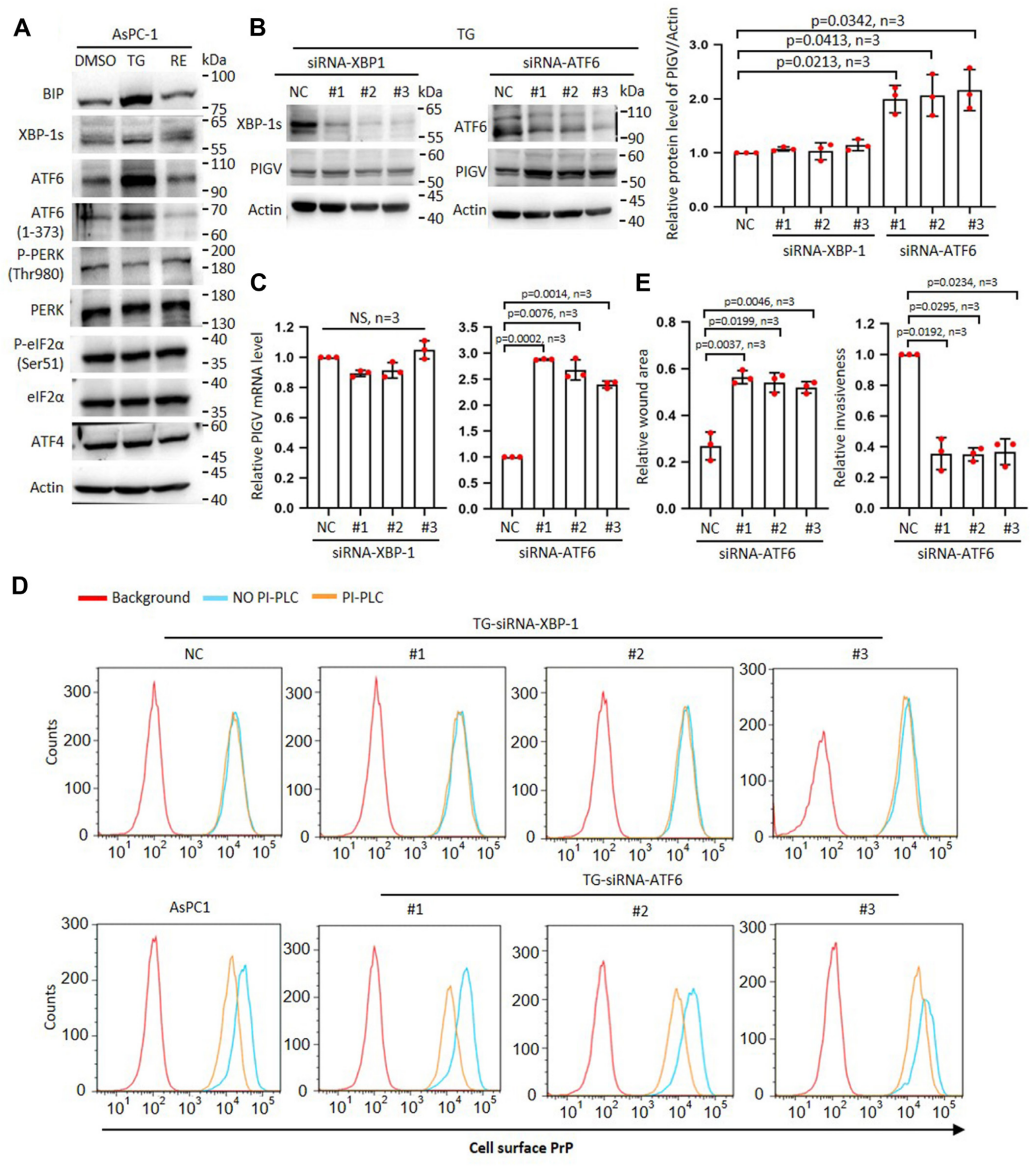
**Tg treatment increases ATF6 to reduce PIGV, resulting in the accumulation of pro-PrP.***A*, immunoblot analysis of BiP, XBP-1s, ATF6, c-ATF6, p-PERK, PERK, p-eIF2α, eIF2α, and ATF4 for cell lysates made from DMSO, TG, and RE AsPC-1 cells. *B*, immunoblot analysis of XBP-1s, ATF6, and PIGV (*left panels*) and statistical analysis (*right panel*) of PIGV after siRNA silencing of XBP-1 and ATF6 in TG cells. *C*, statistical analysis of QPCR results of the effects of XBP-1 and ATF-6 silencing on PIGV mRNA in TG cells. *D*, flow cytometry analysis of the effects of XBP-1 and ATF-6 silencing on the sensitivity of cell surface PrP to PI-PLC treatment. *E*, statistical analysis of wound healing and Matrigel results of ATF-6 silencing on the migration and invasiveness of TG cells. The data were expressed as mean ± SD and were analyzed by two-tailed double sample heteroscedasticity Student's *t* test. ATF6, activating transcription factor 6; BiP, binding immunoglobulin protein; c-ATF6, cleaved activating transcription factor 6; eIF2α, eukaryotic translation initiation factor 2 alpha; NC, non-targeting siRNA control; NS, not significant; PIGV, phosphatidylinositol-glycan biosynthesis class V protein; PI-PLC, phosphoinositide-specific phospholipase C; PERK, pancreatic ER kinase-like ER kinase; p-eIF2α, phosphorylated eIF2α; PrP, prion protein; Tg, thapsigargin.

Imbalance in proteostasis caused by ER stress can result in unfolded protein response (UPR) (23), which is initiated by three transmembrane protein sensors: inositol-requiring protein 1 alpha (IRE1α), pancreatic ER kinase-like ER kinase (PERK), and activating transcription factor 6 (ATF6) (24). One of the prominent markers of UPR response is the chaperon protein binding immunoglobulin protein (BiP) (25). We therefore investigated whether persistent ER stress induces increased abundance of BiP in AsPC-1 cells. We found that Tg treatment indeed increased the levels of BiP, and the levels of BiP returned to normal control levels 4 days after the removal of Tg (Figs. 3*A*, and S3*A*). In addition, persistent ER stress also induced increased abundance of spliced form of X-box–binding protein 1 (XBP-1s), ATF6 and c-ATF6, but not eukaryotic translation initiation factor 2 alpha (eIF2α), phosphorylated-eIF2α, p-PERK, PERK, and ATF4 (Figs. 3*A* and S3*A*).

Next, we attenuated ATF6 and XBP-1 individually by siRNA in AsPC-1-Tg cells to investigate the effects on PIGV and PrP (Figs. 3*B* and S3*B*). The results showed that silencing ATF6 but not XBP1 increased the level of PIGV mRNA and its protein levels (Fig. 3, *B* and *C*). In addition, silencing ATF6 but not XBP1 increased the sensitivity of cell surface PrP to PI-PLC (Figs. 3*D* and S3*C*). We further analyzed the effects of ATF-6 silencing on the migration and invasion of TG cells. As expected, silencing ATF6 significantly decreased the mobile and invasive capability of TG cells (Figs. 3*E* and S3*D*).

### The accumulation of pro-PrP is induced by Tg *via* c-ATF6

**Figure 4:**
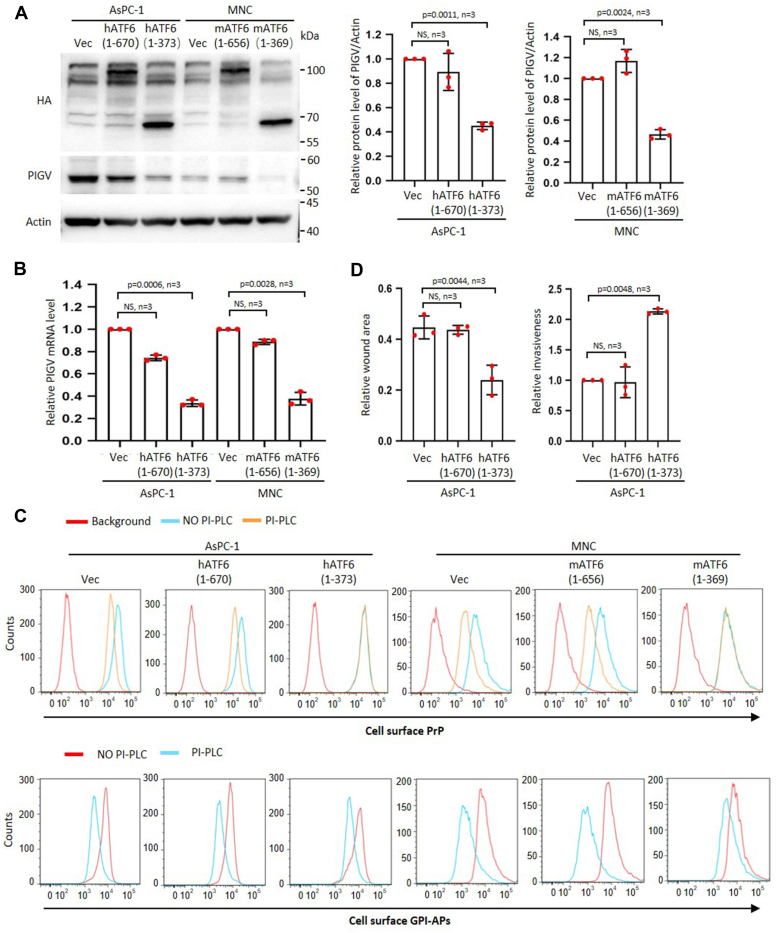
**The accumulation of pro-PrP is induced by Tg *via* c-ATF6.***A*, immunoblot analysis of ATF6 and PIGV of AsPC-1 and MNC after expressing full-length ATF6 or c-ATF6 (*left*). Statistical analysis of PIGV immunoblotting results (*right panels*). *B*, statistical analysis of QPCR results of PIGV mRNA levels after expressing full-length ATF6 or c-ATF6 in AsPC-1 and MNC. *C*, flow cytometry analysis of the effects of expressing full-length ATF6 or c-ATF6 in AsPC-1 and MNC on the sensitivity of cell surface PrP (*top panels*) or GPI-APs to PI-PLC treatment. *D*, statistical analysis of wound healing and Matrigel analysis of expressing full-length ATF6 or c-ATF6 on the migration and invasiveness of AsPC-1 cells. The data were expressed as mean ± SD and were analyzed by two-tailed double sample heteroscedasticity Student's *t* test. ATF6, activating transcription factor 6; c-ATF6, cleaved activating transcription factor 6; GPI, glycosylphosphatidylinositol; GPI-AP, GPI-anchored protein; MNC, mouse neural cell line; NS, not significant; PIGV, phosphatidylinositol-glycan biosynthesis class V protein; PI-PLC, phosphoinositide-specific phospholipase C; PrP, prion protein; Tg, thapsigargin; Vec, empty vector control.

During Tg-induced ER stress, ATF6 is cleaved in Golgi apparatus to become an active transcription factor (26). To investigate if c-ATF6 is responsible for regulating the transcription of PIGV, we overexpressed an HA-tagged full-length or c-ATF6 in AsPC-1 cell and mouse neuron (designated as AsPC-1-hATF6 and MNC-mATF6, respectively) (Fig. 4*A*). We found that the expression of c-ATF6 but not full-length ATF6 significantly decreased the transcripts and protein levels of PIGV (Fig. 4, *A* and *B*). We further assessed the effects of expressing a full-length ATF6 and a c-ATF6 on the sensitivity of cell surface PrP and GPI-APs to PI-PLC treatment. The results showed that only c-ATF6 expression led to resistance of cell surface PrP to PI-PLC treatment (Figs. 4*C* and S4*A*). In contrast, sensitivity of total cell surface GPI-APs was not obviously affected by the expression of either full-length ATF6 or c-ATF6 (Figs. 4*C* and S4*A*).

We also investigated whether expressing c-ATF6 affected cancer cell migration and invasion *in vitro*, and we found that expressing c-ATF6 but not full-length ATF6 significantly enhanced cancer cell migration and invasion (Figs. 4*D* and S4*B*).

### Hsa-miR-449c-5p suppresses PIGV mRNA level

C-ATF6 is a transcription factor that potentially can bind the promoter region of a gene to activate or repress its transcription; an increase in the expression of c-ATF6 reduces the level of PIGV. Thus, we first investigated if c-ATF6 functions as a transcription repressor of PIGV by the reporter assays. Unexpectedly, expressing neither full-length nor c-ATF6 in AsPC-1 cells with the reporter containing the promoter region of PIGV resulted in a significant reduction in luciferase activity (Fig. S5*A*), suggesting that c-ATF6 does not behave as a repressor for PIGV.

**Figure 5:**
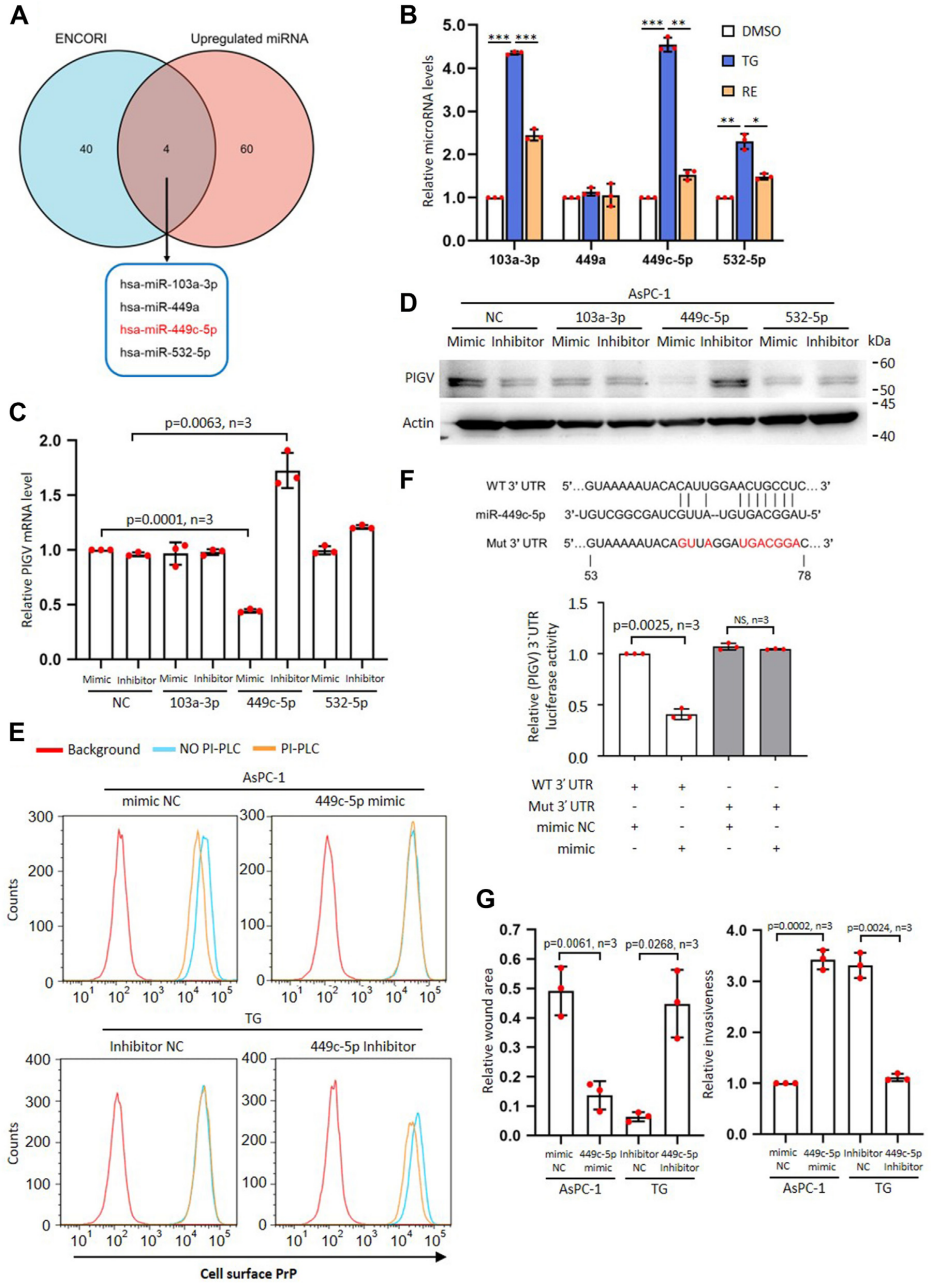
**Hsa-miR-449c-5p suppresses PIGV mRNA level.***A*, diagram to show the number of shared microRNAs using ENCORI program predicting microRNAs regulating PIGV and deep sequencing of microRNAs with increased abundance in Tg-treated AsPC-1 cells. *B*, statistical analysis of QPCR results of microRNAs 103a-3p, 449a, 449c-5p, and 532-5p levels in DMSO, TG, and RE AsPC-1 cells. *C*, statistical analysis of QPCR results of the effects of mimic or inhibitor of microRNAs 103a-3p, 449c-5p, and 532-5p treatment on the mRNA levels of PIGV in AsPC-1 cells. *D*, immunoblot analysis of the effects of mimic or inhibitor of microRNAs 103a-3p, 449c-5p, and 532-5p on the protein levels of PIGV in AsPC-1 cells. *E*, flow cytometry analysis of the sensitivity of AsPC-1 or TG cell surface PrP to PI-PLC treatment when cells are treated with 449c-5p mimic or inhibitor, respectively. *F*, luciferase activity assay of PIGV 3′-UTR (diagram row 1) or mutated 3′-UTR (diagram row 3) when 293T cells are treated with mimic or inhibitor of microRNA449c-5p or control. *G*, statistical analysis of wound healing and Matrigel results of mimic or inhibitor of microRNA449c-5p treatment on the migration and invasion of AsPC-1 or TG cells, respectively. The data were expressed as mean ± SD and were analyzed by two-tailed double sample heteroscedasticity Student's *t* test. ∗*p*< 0.05, ∗∗*p*< 0.01 and ∗∗∗*p* < 0.001. NC, non-targeting miRNA mimic or inhibitor control; NS, not significant; PIGV, phosphatidylinositol-glycan biosynthesis class V protein; PI-PLC, phosphoinositide-specific phospholipase C; PrP, prion protein; Tg, thapsigargin.

Next, we posited that a decrease of PIGV transcripts induced by c-ATF6 might be a result of epigenetic regulation, such as by miRs. We performed miR sequencing for AsPC-1, TG, RE cells. Sixty-four miRs were significantly increased in TG compared to AsPC-1, and these 64 miRs were significantly decreased in RE cells (Fig. S5*A* and Table S1). We then employed the ENCORI program (27) to predict miRs that target PIGV. We found that 44 miRs could target PIGV (Fig. 5*A* and Table S2), among which four increased miRs were found in the 64 miRs enhanced in the TG cells (Fig. 5*A*). Next, we investigated whether the four miRs were increased in abundance in TG but decreased in RE cells by qPCR. The results showed that the expression of miRs 103a-3p, 449c-5p, and 532-5p but not 449a satisfied our hypothesis (Fig. 5*B*).

The major functions of miRs are to reduce mRNA stability or repress mRNA translation. We first focused on the miRs 103a-3p, 449c-5p, and 532-5p to investigate how their expression affects PIGV transcripts by either silencing the miRs or using mimic miRs in AsPC-1 cells. The results showed that only inhibitor of miR 449c-5p significantly enhanced PIGV transcripts, while mimic of 449c-5p significantly suppressed PIGV transcripts (Fig. 5*C*). Immunoblot analysis further confirmed that the inhibitor of miR 449c-5p increased the protein level of PIGV, while the mimic of miR 449c-5p decreased the protein level of PIGV (Figs. 5*D* and S5*B*). We then assessed the effects of miR 449c-5p inhibitor or mimic on the sensitivity to PI-PLC treatment of AsPC-1 and TG cells. We found that miR 449c-5p mimic treatment resulted in PI-PLC resistance of PrP on AsPC-1 cell surface, while miR 449c-5p inhibitor caused PI-PLC sensitivity of PrP on TG cell surface (Figs. 5*E* and S5*C*). To investigate how miR 449c-5p suppressed PIGV level, we performed the reporter assays in the 293T cells by treating the cells either transfected with a WT PIGV-3′UTR or a mutated PIGV-3′UTR with miR 449c-5p (Fig. 5*F*). In the mutated PIGV-3′UTR, the miR 449c-5p-binding site was lost. The results showed that miR 449c-5p mimic could repress the luciferase activity of WT PIGV 3′-UTR but not the mutated one (Fig. 5*F*). Finally, we assessed the effects of miR 449c-5p inhibitor or mimic on the migration and invasiveness of AsPC-1 or TG cells. We found that miR 449c-5p mimic significantly enhanced the migration and invasion of AsPC-1 cells (Figs. 5*G* and S5*D*). In contrast, miR 449c-5p inhibitor significantly decreased the migration and invasion of TG cells (Figs. 5*G* and S5*D*). Thus, ER stress–induced miR 449c-5p suppresses the mRNA of PIGV, disrupting the GPI modification process and leading to the accumulation of pro-PrP, which confers migration and invasion advantages of AsPC-1 cells.

### C-ATF6 decreases PIGV mRNA level *via* hsa-miR449c-5p

**Figure 6:**
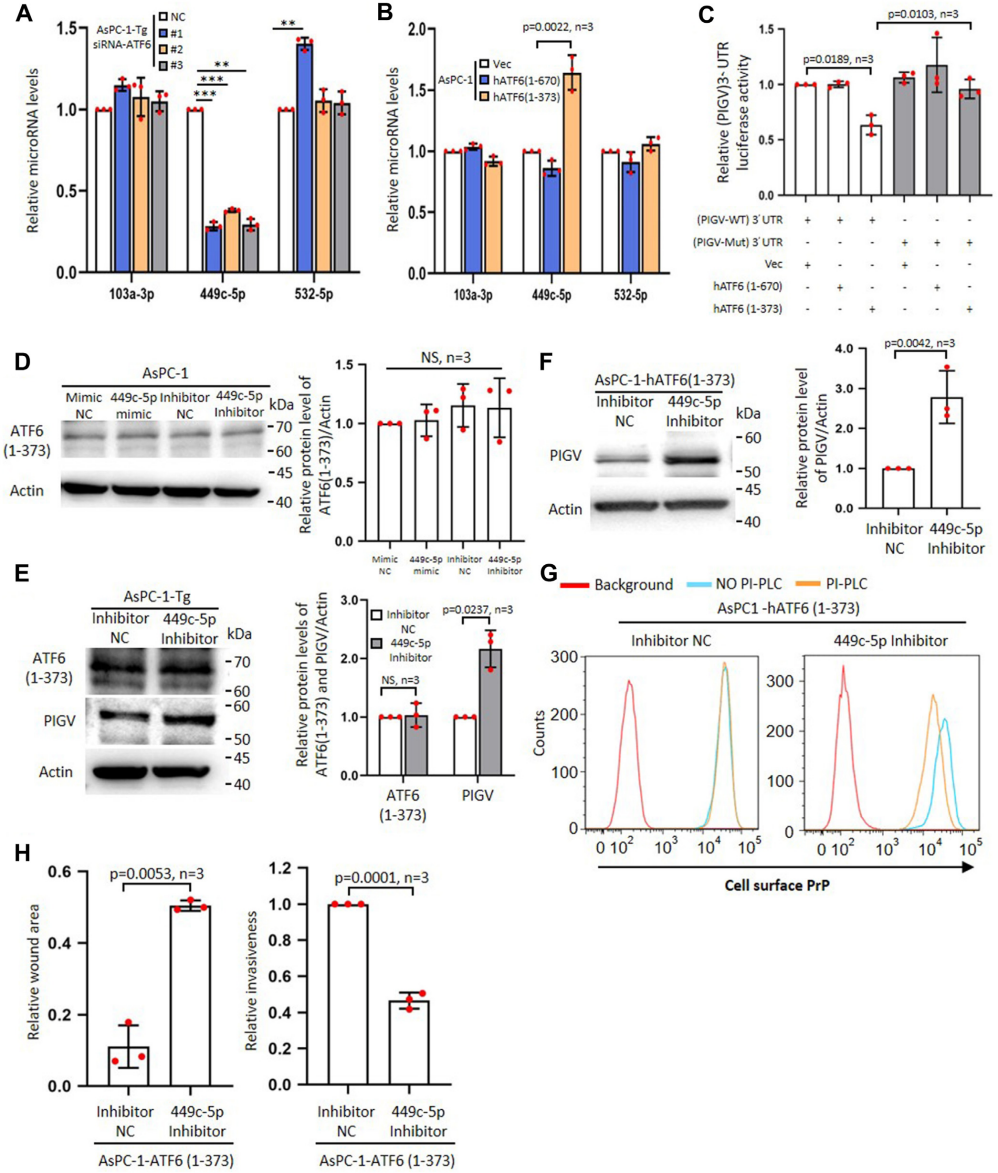
**C-ATF6 inhibits PIGV mRNA level *via* hsa-miR449c-5p.***A*, statistical analysis of QPCR results of silencing ATF6 in AsPC-1-Tg on the levels of microRNAs 103a-3p, 449c-5p, and 532-5p. NC, nontargeting siRNA control. *B*, statistical analysis of QPCR results of expressing full length or c-ATF6 in AsPC-1 on the levels of microRNAs 103a-3p, 449c-5p, and 532-5p. *C*, statistical analysis of luciferase activity assay of PIGV 3′-UTR or mutated 3′-UTR in 293T cells expressing full length or c-ATF6. *D*, immunoblot analysis of c-ATF6 from AsPC-1 cells treated with inhibitor or mimic of microRNA449c-5p. AsPC-1 cells are also treated with inhibitor or mimics of control microRNA (*left panel*). Statistical analysis of immunoblot results is shown (*right panel*). NC, nontargeting miRNA mimic or inhibitor control. *E*, immunoblot analysis of c-ATF6 and PIGV from AsPC-1-Tg cells treated with inhibitor of microRNA449c-5p (*left*). Statistical analysis of immunoblot results is shown (*right panel*). *F*, immunoblot analysis of PIGV from AsPC-1 cells expressing c-ATF6 and treated with inhibitor of microRNA449c-5p or nontargeting miRNA control (NC) (*left*). Statistical analysis of the immunoblots is shown (*right*). *G*, flow cytometry analysis of sensitivity of c-ATF6 expressing AsPC-1 cell surface PrP to the treatment of PI-PLC after the cells are treated with inhibitor of microRNA449c-5p. *H*, statistical analysis of wound healing and Matrigel results of inhibitor of microRNA449c-5p on the migration and invasion of AsPC-1 cells expressing c-ATF6. The data were expressed as mean ± SD and were analyzed by two-tailed double sample heteroscedasticity Student's *t* test. ∗∗*p*< 0.01 and ∗∗∗*p* < 0.001. ATF6, activating transcription factor 6; c-ATF6, cleaved activating transcription factor 6; NS, not significant; PrP, prion protein; PIGV, phosphatidylinositol-glycan biosynthesis class V protein; PI-PLC, phosphoinositide-specific phospholipase C; Tg, thapsigargin; Vec, empty vector control.

To investigate whether the increased abundance of miR 449c-5p was induced by ER stress *via* c-ATF6, we quantified relative miR449c-5p, miR103a-3p, and 532-5p levels in ATF-6–silenced TG cells. The results showed that silencing ATF6 significantly mitigated the levels of miR 449c-5p but not miR103a-3p and 532-5p (Fig. 6*A*). To confirm that the level of miR 449c-5p was promoted by c-ATF6, we quantified its level in full length or c-ATF6–expressing AsPC-1 cells (designated as AsPC-1-hATF6) (Fig. S6*A*). The results showed that c-ATF6 but not full-length ATF6 increased the level of miR449c-5p but not miR103a-3p or 532-5p (Fig. 6*B*). Additional evidence that c-ATF6 but not full-length ATF6 could reduce PIGV 3′-UTR came from the luciferase reporter assays. In these assays, c-ATF6 but not full-length ATF6 could suppress the luciferase activity of WT PIGV-3′UTR (Fig. 6*C*). However, c-ATF6 could not suppress the luciferase activity of a mutant PIGV-3′UTR that could not bind miR 449c-5p (Fig. 6*C*). These results suggest that c-ATF6 is upstream of miR 449c-5p, and PIGV level is mitigated by miR 449c-5p. To further prove this point, we treated AsPC-1 cells with miR 449c-5p inhibitor or mimic, respectively. The results showed that the treatment did not affect ATF6 mRNA and protein levels in AsPC-1 cells (Figs. 6*D* and S6*B*). We also treated TG cells with miR 449c-5p inhibitor. The results showed that the treatment increased the mRNA and protein levels of PIGV but not c-ATF6 in TG cells (Figs. 6*E* and S6*C*). To establish that c-ATF6 is upstream of miR 449c-5p and inhibits PIGV abundance *via* miR 449c-5p, we treated AsPC-1-hATF6 (1–373) cells with a miR 449c-5p inhibitor and detected the effects on PIGV and PrP. The results showed that inhibiting the function of miR 449c-5p was enough to prevent the c-ATF6 in decreasing the level of PIGV (Fig. 6*F*) and the sensitivity of PrP to PI-PLC treatment (Figs. 6*G* and S6*D*). Accordingly, such a treatment also inhibited the migration and invasion of AsPC-1-hATF6 (1–373) cells (Figs. 6*H* and S6*E*).

### ATF6–miR449c-5p–PIGV axis also contributes to PDAC in humans and are makers of prognosis in patients with PDAC

**Figure 7:**
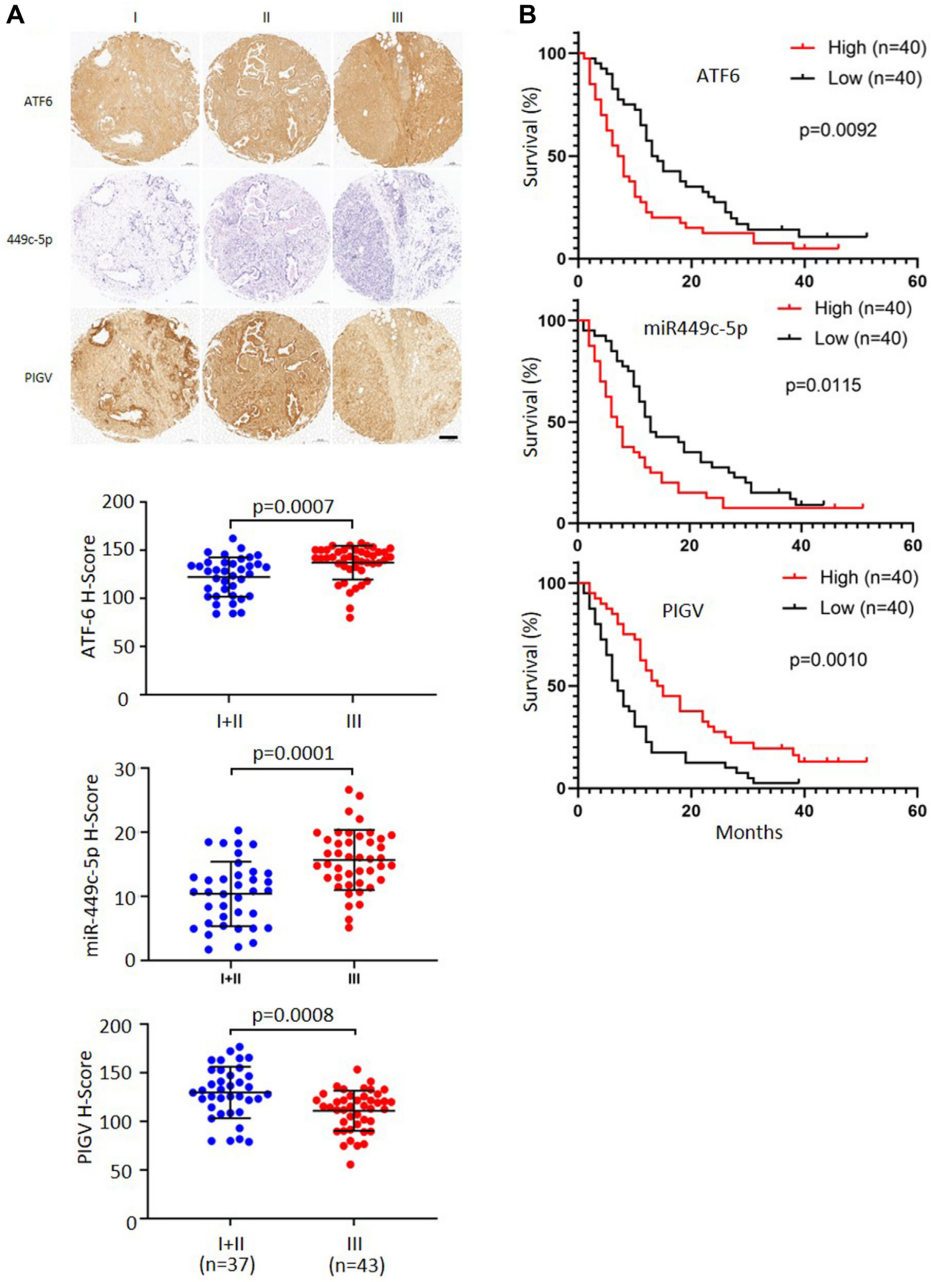
**ATF6–miR449c-5p–PIGV axis contributes to PDAC in humans and marks prognosis in patients with PDAC.***A*, immunohistochemical analysis of ATF6 and PIGV and *in situ* hybridization analysis of microRNA449c-5p expression in different stages from matched PDAC biopsies (*upper panels*). Statistical results of the immunohistochemical and *in situ* hybridization results between stage I+II and stage III PDAC are shown (*bottom panels*). Scale bars represent 200 μm. *B*, Kaplan–Meier survival plot of PDAC patients based on the immunohistochemical and *in situ* hybridization analysis results. *p*-values and number of biopsies are indicated. The statistical significance of prognosis analysis was assessed using the Mantel-Cox test. The other data were expressed as mean ± SD and were analyzed by two-tailed double sample heteroscedasticity Student's *t* test. ATF6, activating transcription factor 6; PDAC, pancreatic ductal cell adenocarcinoma; PIGV, phosphatidylinositol-glycan biosynthesis class V protein.

To investigate the clinical relevance of the ATF6–miR449c-5p–PIGV axis in human PDAC tumorigenesis, we first performed a prognosis analysis for patients with PDAC (https://kmplot.com/analysis/) (28). The results showed that higher expressions of ATF6 and miR 449c-5p were significantly correlated with poorer prognosis of patients with PDAC (Fig. S7*A*). We then performed immunohistochemical staining of ATF6 or PIGV or *in situ* hybridization of miR 449c-5p on tissue arrays prepared from PDAC patients. The results showed that in comparison to matched paratumor tissues, tumors had significantly lower levels of PIGV (Fig. S7*B*). In comparison, there were no significant differences between tumor and paratumor for levels of miR449c-5p and ATF6 (Fig. S7*B*). Furthermore, the levels of ATF-6 and miR449c-5p were significantly elevated in stage III PDAC than those in stages I+II PDAC (Fig. 7*A*). Accordingly, the levels of PIGV were significantly decreased in stage III PDAC than those in stages I+II PDAC (Fig. 7*A*). Moreover, a higher level of ATF6 and miR449c-5p and a lower level of PIGV were markers for poorer prognosis of patients with PDAC (Fig. 7*B*). Thus, ER stress–induced pro-PrP *via* ATF6–miR449c-5p–PIGV axis also contributes to PDAC progression in human.

## Discussion

Here, we provide evidence showing that the cause of the accumulation of pro-PrP in PDAC and MNC is due to persistent ER stress and UPR. ER stress increases the abundance of ATF6 (Fig. 3). Upon cleavage at the Golgi, a c-ATF6 is generated, and instead of functioning as a transcriptional repressor, it increases *miR-449c-5p* abundance (Figs. 6 and S5). The miR then reduces the level of PIGV mRNA (Figs. 5 and 6). A reduction in PIGV protein disrupts the GPI modification process (Figs. 2 and S2), resulting in the conversion of GPI-anchored PrP into pro-PrP, rendering the tumor cells more aggressive. We are aware that our conclusion is solely based on studying of one PDAC cell line, AsPC-1. However, the fact that this pathway is recapitulated in human PDAC biopsy samples and has prognostic values implies that this axis is also faithfully operating in human PDAC biology (Fig. 7). Additionally, this axis also operates in an MNC (Figs. 1, 2 and 4), further supporting this axis may be conserved under ER stress. Since persistent ER stress is also a hallmarker of many neurodegenerative diseases, whether such a conversion contributes to neurodegenerative diseases, in which PrP has been implicated deeply, warrants further investigation.

A plethora of evidence supports that PrP plays an important role in tumorigenesis. Higher abundance of PrP has been detected in a variety of human cancers, and higher expression has been correlated with poorer prognosis in patients with PDAC, gastric cancer, melanoma, and colon cancer (8, 11, 29, 30, 31, 32, 33, 34, 35, 36, 37). The reported mechanisms of PrP contributing to tumorigenesis span from enhanced growth advantages and metastasis to increased drug resistance to elevated stemness of cancer cells (31, 38, 39, 40, 41).

At the mechanistic levels, two events must happen in the transformation of pancreatic ductal cells prior to the development of full-blown PDAC; the increased abundance of PrP and the failure to remove the GPI-PSS. ER stress induced in our model appears to disrupt the GPI anchor modification process by only causing the accumulation of pro-PrP without increasing the expression levels of PrP (Fig. S1*E*), and the mean fluorescent intensities of cell surface PrP are also comparable between AsPC-1, TG, and RE cells (Fig. 1, *A*–*C* and *E*).

Chemicals, such as Tg, BFA, tunicamycin, and DTT have long been used to artificially induce ER stress in cell models (18, 41, 42, 43). Under acute ER stress (with Tg concentration around 100 nM) or chronic ER stress (with Tg concentration less than 2 nM), GPI-APs show different responses (18, 42). Acute ER stress caused degradation of misfolded GPI-APs in the lysosome, which may be protein-specific or cell context–dependent, with very little GPI-anchored protein on the cell surface (42, 44, 45). In comparison, persistent ER stress results in the transit of inositol-acylated GPI-APs to the cell surface (18). However, in our cell model, the PrP detected on the cell surface is a pro-PrP instead of an inositol-acylated GPI-anchored PrP (Fig. 1, *A* and *D*). The reason for this discrepancy is not clear. The nature of the GPI-APs being studied and the use of different cell types or experimental conditions may contribute to the observed differences.

More and more evidences have shown that stressors enriched in the tumor microenvironment cause ER stress in cancer cells and in stromal cells (46). In addition, cancer cells can transfer ER stress to paratumor cells inside tumor microenvironment (47, 48, 49). It is thus expected that there may be no significant difference in ATF6 and miR449c-5p levels between tumor and paratumor (Fig. S7*B*). In contrast, there is a significant difference in PIGV levels between tumor and paratumor, suggesting that ATF6-miR449c-5p–decreased PIGV expression is tumor cell–dependent. The exact mechanism warrants further investigation. Losses of chromosome 1p36 in which the PIGV gene is located have been linked to a broad range of human malignancies (50, 51, 52, 53). It is conceivable that loss of PIGV-induced pro-PrP may contribute to the progression of the malignant tumors. It is almost certain that a reduction in PIGV level will impact the synthesis of GPI-APs other than PrP. Changes in some of these proteins may have biological consequences. However, in PDAC, the effects on PrP appear to be the most prominent. “Knocking out” PrP completely eliminated the phenotypes, such as increases in migration and invasion seen in TG cells (Fig. 1*G*).

Even though c-ATF6 is known to be a transcription factor in TG cells, c-ATF6 works by enhancing the expression levels of miRNA 449c-5p. The mechanism by which c-ATF6 enhances miRNA 449c-5p level is not clear. c-ATF6 is reported to bind to a consensus motif in the promoter regions of its target genes (54). But this motif is absent in the promoter region of miR 449c-5p. Previously, ATF6 branch has been shown to regulate miR expression (55). In addition, ATF6 can mediate activation of mammalian target of rapamycin (56), which has been shown to regulate miR expression (57). Whether or not this pathway is involved in ER stress–induced pro-PrP accumulation warrants further investigation.

Inflammation is an important contributing factor to tumorigenesis. Inflammation is known to increase the expression of PrP (58, 59, 60). Since ER stress has been shown to be induced by inflammatory cytokines inside the tumor microenvironment (61, 62), local inflammation in the pancreas, such as pancreatitis, may be a predisposing contributing factor for increase PrP expression as well as generating pro-PrP in precancerous ductal cells as reported (8). In addition, PDAC cell is covered by a dense matrix which can cause hypoxia, a potent inducer of ER stress also shown to induce the expression of PrP (63, 64). Hence, higher abundance of PrP and the accumulation of pro-PrP are expected as PDAC cells develop, which potentializes the high metastasis of PDAC. Under persistent ER stress, the conversion of a GPI-anchored PrP into pro-PrP in AsPC-1 cells is reversible when the inducer is removed (Fig. 1). This seems in contradictory to what we have observed that most PDAC cell lines express pro-PrP (8, 11). However, we believe that mutation(s) or irreversibly reduction of enzymes involved in GPI-anchor synthesis occur in a later stage which thus maybe an accomplice fixed during cancer progression.

In summary, we have identified a conserved axis for the conversion of a GPI-anchored PrP into pro-PrP under persistent ER stress, which explains the occurrence of pro-PrP in most PDAC cells and why patients whose tumor express higher level of PrP have poorer prognosis.

## Experimental procedures

### Cell lines

AsPC-1 and HEK293T cell lines were purchased from American Type Culture Collection. MNC-mPrP cells were generated from *Prnp*-KO mouse neuron as described (9). AsPC-1 and AsPC-1-*PRNP* ^−/−^ cells were cultured in RPMI1640 medium (31800-022) supplemented with heat-inactivated 10% fetal bovine serum (FBS) (10099-141, Gibco), 1.5 g/L sodium bicarbonate, 4.5 g/L glucose, 3 g/L Hepes (V900477, Sigma-Aldrich), 1 mmol/L sodium pyruvate (11360-070, Gibco), 1% GlutaMAX-1 (35050-061, Gibco), 1% antibiotic penicillin–streptomycin solution (03-031-1, Biological Industries). MNC-mPrP and HEK293T cells were cultured in high glucose Dulbecco's modified Eagle's medium (Gibco) supplemented with heat-inactivated 10% FBS, 1% penicillin–streptomycin solution. All cell lines used are authentic and have been tested *Mycoplasma* free. The cells were maintained in a 37 °C, 5% CO2, 95% humidity incubator. To develop persistent ER stress cell model, AsPC-1 or MNC-PrP cells were cultured in their media containing 30 nM Tg (T9033, Sigma-Aldrich) or 70 nM BFA (B5936, Sigma-Aldrich) for more than 14 days and maintained under the same condition after pro-PrP has been formed. These cells were designated as AsPC-1-Tg (TG), AsPC-1-BFA, or MNC-Tg, respectively. The AsPC-1-Tg/BFA cells were cultured in the media removal Tg/BFA for more than 4 days and were designated as AsPC-1-Tg/BFA-RE (RE).

### Antibodies and reagents

Antibodies (Abs) against PrP (4H2 and 8B4) were generated in our laboratory as described (9). Abs against HA-tag (3724), BiP (3177), XBP-1s (40435), phospho-PERK (3179), PERK (5683), phospho-eIF2α (p-eIF2a, 3398), eIF2α (5324), and ATF4 (11815) were purchased from Cell Signaling Technology. Ab against PIGV (ab237692) was purchased from Abcam. Ab against Glypican-1 (sc-101827) was purchased from Santa Cruz Biotechnology. Ab against Thy-1 (hb-8553) was purchased from American Type Culture Collection. Ab against ATF6 (NBP1-40256) was purchased from Novus Biologicals. Abs against PIGZ (17384-1-AP) and GDPD5 (25703-1-AP) were purchased from Proteintech. Ab against actin (KM9001) was purchased from Tianjin Sungene Biotech. Mouse IgG1 (400165) was purchased from Biolegend. Horseradish peroxidase (HRP)-conjugated goat anti-mouse IgG secondary antibody (AS003) and HRP-conjugated goat anti-rabbit IgG antibody (AS014) were purchased from Abclonal. Alexa Fluor 488 nm–conjugated goat anti-rabbit IgG (A-11008) and Alexa Fluor 555 nm–conjugated goat anti-mouse IgG (A-21424) secondary antibody were purchased from Invitrogen. All Abs were used according to the instructions of the manufacturer. Proteinase inhibitor cocktail (11697498001) and 4, 6-diamidino-2-phenylindole (10236276001) were purchased from Roche Diagnostics. 2 × Phanta Max master mix (P515-01) was purchased from Vazyme. Phenylmethylsulfonyl fluoride (PMSF, P7626) and DMSO (D2650) were purchased from Sigma-Aldrich. All reagents purchased from commercial sources were used according to the suppliers’ recommendations.

### Construction of plasmids

Cloning vector PHAGE-CMV-MCS-IZsGreen and the packaging plasmids psPAX2, pMD2.G were kindly provided by Professor Zan Huang (Wuhan University). pHAGE-CMV-MCS-3×HA-IZsGreen was generated in our laboratory (65). The plasmids pHAGE-PIGV-3×HA, pHAGE-PIGZ-3×HA, pHAGE-HA-hATF6 (1-670)-3×HA, pHAGE-HA-hATF6 (1-373)-3×HA, pHAGE-HA-mATF6 (1-656)-3×HA, and pHAGE-HA-mATF6 (1-369)-3×HA were constructed as following. Briefly, PIGV, PIGZ, hATF6 (1-670), and hATF6 (1-373) were amplified from AsPC-1 cells with primers listed in Table S3. mATF6 (1-656) and mATF6 (1-369) were amplified from MNC-mPrP cells with primers listed in Table S3. The PCR-amplified fragment was gel purified and digested with *NotI* and *NheI* at 37 °C for 1 h. The digested samples were further gel purified and ligated into pHAGE-CMV-MCS-3×HA-IZsGreen vector by standard molecular biology techniques. To generate plasmid pGL3-PIGVPRO, PIGV protein promotor sequence (2000 bp of sequence upstream of the transcription start site) was amplified with the primers listed in Table S3 using genomic DNA purified from AsPC-1 cells. The amplified sequences were gel purified and digested with *KpnI* and *MluI* at 37 °C for 1 h. The digested samples were further gel purified and ligated into pGL3-Basic vector (Promega). To generate plasmids pmiR-V3'-UTR (WT) and pmiR-V3'-UTR (MUT), the WT 3'-UTR region of PIGV, which contains the miR-449c-5p–binding site, and a 3'-UTR variant with a mutation at the miR-449c-5p–binding site were synthesized by Tsingke Biotechnology. The sequences were gel purified and digested with *XhoI* and *NotI* at 37 °C for 1 h. The digested samples were further gel purified and ligated into pmiR-RB-Report vector (Ribobio). All the constructs were sequence-confirmed.

### PI-PLC treatment of cells and flow cytometry analysis of cell surface PrP

PI-PLC treatment was performed according to Li *et al* (2023, Star Protocol). Briefly, cells were seeded in 10-cm Petri dishes overnight and then rinsed three times with ice-cold PBS followed by 0.25% trypsin/EDTA treatment. Resuspended cells were collected by centrifugation at 500×*g* for 3 min. To measure PrP levels on cell surface, the sample was equally divided into three parts. One part was treated with PI-PLC (P5542, Sigma-Aldrich) (0.1 unit/ml); the other two samples were mixed with PBS. All samples were kept at 37 °C for 1 h and then rinsed twice with ice-cold PBS followed by staining with 4H2 (10 μg/ml) or IgG1 for 30 min at 4 °C. After rinse with ice-cold PBS six times by centrifugation at 500*g* for 3 min, Alexa Fluor 555 nm–conjugated goat anti-mouse IgG Ab (5 ng/ml) were then added and the cells were stained for 30 min at 4 °C in dark. To measure GPI-APs levels on cell surface, one part of cells with and without PI-PLC treatment were incubated with FLAER (Alexa Fluor 488 proaerolysin) (FL1S, Cedarlane) for 30 min at 4 °C in dark. Cells were further rinsed six times and analyzed in a FACS C6 flow cytometer (BD Biosciences) as described (66). For each sample, select the drawing tool in the screen to draw one Dot Plot of omnidirectional and lateral angles, X-axis label is FSC-A, Y-axis label is SSC-A, painting single cell gating to analyze cell population. The number of cells collected is 10,000. The data were then analyzed using FlowJo 7.6 (www.flowjo.com). To calculate the PI-PLC treatment on PrP or GPI-APs, the formula was used as described by (17).

### CPDB treatment of immunoaffinity-purified PrP

CPDB treatment was performed according to Li *et al* (66). Briefly, to purify PrP from various cell lines, fresh cell lysate was prepared and affinity-purified with 100 μg of mAb 8B4 bound to 100 μl of profound co-immunoprecipitated agarose beads (Aminolink, Thermo Fisher Scientific) according to the manufacturer’s instructions. Then, 400 μl of cell lysate was added to the column containing the agarose resin coupling with Abs overnight at 4 °C. The resin was washed six times with ice-cold lysis buffer at 4 °C by centrifuging at 1000*g* for 1 min. After that, 100 μl of elution buffer was added. After 5 min incubation at room temperature (RT), PrP was eluted by centrifuging at 1000*g* for 1 min and quickly neutralized in Tris-glycine (pH9.4), which was then subjected to CPDB treatment (0.5 unit/20 μl of eluted PrP) at RT for different periods of time. The samples were then separated on SDS-PAGE and immunoblotted with 4H2.

### RNA extraction and quantitative real time PCR

To quantify mRNA from drug-treated or DMSO-treated cells, total RNA was extracted using the FastPure Cell/Tissue Total RNA Isolation Kit (RC112-01, Vazyme) as instructed. Briefly, 1 μg of total RNA was reverse-transcribed as complementary DNA using HiScript III first Strand cDNA Synthesis kit (R312-01, Vazyme). qPCR procedure was carried out using ChamQ Universal SYBR qPCR master mix (Q711-02, Vazyme) on a Bio-Rad Connect real-time PCR instrument (CFX Connect TM Optics Module). β-actin was used as a reference gene. Gene-specific primers were as listed in the Table S4. For miRNA detection, total RNA was reverse-transcribed as cDNA and subsequent qPCR analysis was performed using the Bulge-Loop miRNA qRT-PCR Starter Kit (C10211-2, Ribobio). The miRNA qPCR primer sets were purchased from RiboBio (Table S6). U6 snRNA was used as the endogenous control to normalize miRNA expression. The 2-ΔΔCt method was used to calculate fold changes in gene expression.

### RNA-seq and data analysis

Three biological replicates of each sample were used for mRNA and miRNA sequencing. Sequencing libraries of mRNAs were generated using MGIEasy RNA Library Prep Kit (1000006383, MGI Tech) following manufacturer’s instructions, and the sequencing libraries of miRNA were generated using MGIEasy Small RNA Library Prep Kit (940-000196-00, MGI Tech). The libraries were sequenced using a MGISEQ-2000 platform (MGI Tech). Raw data (raw reads) of the fastq format were first processed using SOAPnuke (v1.5.6). For mRNA sequencing, clean reads were obtained by removing adapter-containing reads, poly-N–containing reads, and low-quality reads from the raw data and followed by mapping to the *Homo sapiens* genome sequence (GCF_000001405.39_GRCh38.p13) using HISAT2 (version 2.1.0). For miRNA sequencing, clean reads were obtained by removing low-quality reads, tag with 5′ adapter pollution, tag without 5′ adapter, tag without insert fragment, tag containing poly-A, and tag less than 15 nt; the miRNA reads were aligned to known mature human miRNA sequences using Bowtie (v 2.0.6). The DESeq2 (v1.4.5) (www.bioconductor.org/packages/devel/bioc/html/DESeq2.html) software was used for data normalization and screening of differentially expressed genes. To remove low-repeat differentially expressed genes, the *p*-value was set to be less than 0.05. log2(foldchange) were defined as >1.0 for genes with increased abundance and < −1.0 for genes with decreased abundance. The Dr Tom Multi-Omics Data Mining system (https://biosys.bgi.com) was used for subsequent data analysis.

### Transfection of plasmids, siRNAs, miRNA mimic, and miRNA inhibitor

To generate overexpressed cells, the overexpression plasmids pHAGE-PIGV-3×HA, pHAGE-PIGZ-3×HA, pHAGE-HA-hATF6 (1-670)-3×HA, pHAGE-HA-hATF6 (1-373)-3×HA, pHAGE-HA-mATF6 (1-656)-3×HA, pHAGE-HA-mATF6 (1–369)-3×HA, and the empty vector (negative control) was used. Briefly, 6-7×10^5^ cells were seeded in the 6-well plate for 12 h (h). Two micrograms of plasmid was transfected using Lipofectamine 2000 (11668-019, Thermo Fisher Scientific) according to the manufacturer’s instructions. After 72 h, cells were collected and subsequent experiments were performed. To generate XBP-1, ATF6, PIGV, or PIGZ-silenced cells, siRNA oligos transfected into cells with PepMute transfection reagent (SL100566, SignaGen) according to the manufacturer’s instructions. siRNAs were purchased from Tsighke Biotech, and the sequences of the siRNAs were listed in the Table S5. siRNAs (30 nM) were transfected into cells at 80% confluence in the 6-well plate. After 72 h, cells were collected and the expression of mRNA and protein of target genes was detected by qPCR and Western blotting, respectively. To generate miRNA overexpressed or silenced cells, mimic or inhibitor of miRNA hsa-miR-103a-3p, hsa-miR-449c-5p, or hsa-miR-532-5p was transfected into cells with PepMute transfection reagent. Target-specific mimic (30 nM) or inhibitor (90 nM) was transfected into cells when cells reach 80% confluence in the 6-well plate. An equal concentration of a nontargeting control sequence was added to experimental samples as controls for non–sequence-specific effects in miRNA experiments. All miRNA mimics and inhibitors, including nontargeting miRNA, were purchased from RiboBio (Table S7).

### SDS-PAGE and Western blotting assays

Other than indicated, cell lysates were made in lysis buffer (20 mM Tris–HCl (pH 7.5), 150 mM NaCl, 1 mM EDTA, 1 mM EGTA, 1% Triton X-100, 2.5 mM sodium pyrophosphate, 1 mM β-glycerol phosphate, 1 mM PMSF, 1 mM Na3VO4, and a protease inhibitor cocktail) as described (8). For alkaline phosphatase (AP) treatment, cell lysate was made in the NP40 lysis buffer (20 mM Tris–HCl (pH 7.5), 150 mM NaCl, 1 mM EDTA, 1% NP40). Briefly, cells were collected in lysis buffer with protease inhibitors cocktail and PMSF on ice for 20 min. After centrifugation at 13,000*g* for 10 min (mins), protein concentration was quantified by G250 solution. Cell lysates were then mixed with 4 × SDS loading buffer, heated at 100 °C for 10 min, and subjected to 7.5%-12.5% SDS-PAGE. After gel electrophoresis, separated proteins were transferred to 0.45 μm nitrocellulose membrane (Merck Millipore) and blocked with 3% bovine serum albumin (BSA) in Tris buffered saline with 0.1% Tween-20 at RT for 2 h. Nitrocellulose membrane was further incubated with primary Abs at 4 °C overnight. Bound primary Abs were detected by corresponding HRP-conjugated secondary Abs. All immunoblots were quantified according on densitometry using the Image J (imagej.nih.gov/ij/) software (NIH).

### Immunofluorescence staining

Cells were seeded on poly L-lysine–coated glass-bottomed dishes (NEST) overnight and fixed in 4% (W/V) paraformaldehyde for 10 min at RT. Cells were then permeabilized with 0.5% Triton X-100 in PBS for 10 min. After blocking with 1% BSA in PBS with 0.5% Tween-20 (PBST) for 2 h at 25 °C, cells were incubated with an anti-HA Ab (100 ng/μl) overnight at 4 °C. Cells were then rinsed six times with PBST. Bound Abs were detected with the Alexa Fluor 488 nm–conjugated goat anti-rabbit IgG (1:2000) for 1 h at RT in dark. Nuclei were counterstained with 4′,6-diamidino-2-phenylindole (500 ng/ml) for 5 min. Cells were washed six times with PBST. The images were taken by a confocal microscopy (UltraView Vox confocal microscope, PerkinElmer).

### Luciferase reporter assays

The plasmid PHAGE-CMV-MCS-IzsGreen, pHAGE-HA-hATF6 (1-670)-3×HA, or pHAGE-HA-hATF6 (1-373)-3×HA (2.0 μg) was cotransfected with reporter vector pGL3-PIGVPRO (2.0 μg) into AsPC-1 cells using Lipofectamine 2000 for each well in a 6-well plate. The plasmid PHAGE-CMV-MCS-IzsGreen, pHAGE-HA-hATF6 (1-670)-3×HA, or pHAGE-HA-hATF6 (1-373)-3×HA (2.0 μg) was cotransfected with reporter vector pmiR-V3'-UTR (WT) or pmiR-V3'-UTR (MUT) (2.0 μg) into HEK293T cells using Lipofectamine 2000 for each well in a 6-well plate. The plasmid pmiR-V3'-UTR (WT) or pmiR-V3'-UTR (MUT) was cotransfected into HEK293T with mimic of miR-449c-5p or mimic-NC using PepMute transfection reagent for each well in a 6-well plate. Cells were harvested after 48 h, and cell lysates were collected with 1×PLB buffer for firefly and renilla luciferase activities using the dual-luciferase reporter assay system (Promega) according to the manufacturer’s protocol.

### Cell migration assays

Cells were cultured in a 6-well plate until 100% confluency. Wound was inflicted with a tip-cut 200 μl pipette tip, and the supernatant was discarded. The wounds were imaged at 0 and 48 h post wound infliction using an inverted microscopy. Wound area was defined as pixels measured with IMAGE J software as following: the wound area pixels at 48 h post wound infliction/pixels from time zero. Cell migration was calculated as described (11).

### Cell invasion assays

Invasion of cancer cells was determined using 24-well transwell plates (8 μm pore diameter; Corning). Matrigel diluted in culture medium without FBS (100 μl) was added to the upper chamber at 37 °C for 1 h. Cells were serum starved for 24 h before making single cell suspension. Single cell suspension containing 1×10^5^ cells was seeded in the upper chamber with 100 μl culture medium without FBS. The lower chamber was filled with 750 μl culture medium with FBS. Cells in the transwell were cultured for an additional 48 h before cancer cells in upper chamber were scraped with a Q-tip. After fixation (4% paraformaldehyde), cells at the bottom chamber were stained with 0.1% crystal violet for 30 min and rinsed with running water. For each chamber, three fields were randomly pictured at 10 × on an inverted microscopy and cells were counted by Image J (NIH) software. Relative invasive cells are defined as follows: invasive cell counts in reagent-treated cells/invasive cell counts in control-treated cells, which is defined arbitrarily as 1.0.

### Cell proliferation assays

To assay the cytotoxicity of Tg treatment on AsPC-1 cells, 2000 cells were seeded per well in a 96-well plate and cultured with different concentrations of Tg. Twenty microliters of MTS reagent (Promega) was then added to each well 12 h later. The cells were further incubated with MTS reagent at 37 °C for 2 h. OD values were recorded every 24 h at 490 nm until 72 h post cell seeding using enzyme-labeled instrument (Thermo Fisher scientific, Varioskan Lux).

### Histological analysis

For ATF6 and PIGV immunohistochemistry, PDAC tissue microarrays (PAC1602, Superbiotek) were heated at 65 °C for 1 h and then deparaffinized in xylene and alcohol, antigen retrieval with 0.01 M sodium citrate buffer (SSC) (pH 6.0) at 98 °C for 10 min, followed by 3% hydrogen peroxide treatment for 15 min at RT to block endogenous peroxidase activity. The sections were next blocked with 5% BSA in PBST for 1 h. Primary Ab (ATF6 (1:50); PIGV (1:200)) was incubated with the sections overnight at 4 °C in a humidified chamber and then rinsed six times with PBST. HRP-conjugated secondary Ab was then applied to detect bound primary Abs for an additional 45 min at RT. Bound secondary Abs was detected by diaminobenzidine, and the reaction was stopped by immersion of tissue sections in distilled water once brown color appeared. Tissue sections were counterstained by hematoxylin and were dehydrated in graded ethanol. All immunohistochemistry results were examined and determined by AIPATHWELL software (An artificial intelligence learning digital pathological image analysis software developed by Wuhan Servicebio Technology Co) (www.servicebio.cn) using histochemistry score (H-score) (H-score = (percentage of weak intensity × 1) + (percentage of moderate intensity × 2) + (percentage of strong intensity × 3) to determine the overall percentage of ATF6 or PIGV positivity across the entire stained tumor sample, yielding a range from 0 to 300. The image information was collected using a Pannoramic MIDI system (3DHISTECH).

### *In Situ* hybridization

To evaluate relative expression levels of miR-449c-5p in PDAC tissue microarray, in situ hybridization (ISH) was performed. Double-Dig–labeled probe for miR-449c-5p (miRCURY LNA Detection probe, 5′-DIG– and 3′-DIG–labeled hsa-miR-449c-5p, Qiagen) and miRCURY LNA miRNA ISH buffer set (FFPE) (339450, Qiagen) were used. ISH was performed according to manufacturers’ instructions. In brief, tissue microarray was deparaffinized in three washes of xylene for 5 min each and then hydrated through sequentially decreasing ethanol solutions from 100% to 70%. The target demasking step was carried out in Proteinase K solution at a concentration of 0.75 μl/ml at 37 °C for 10 min, following predigestion that allows the access of double-DIG–labeled LNA probes to hybridize to the miRNA sequence. Probe, denatured at 90 °C for 4 min, was diluted in 1x hybridization solution to a final concentration of 40 nM. The slide was covered with SecureSeal hybridization chambers (GBL623504, Grace Bio-Labs) and hybridized at 54 °C for 60 min. The slide was passed through stringent washing steps of 5×, 1× and 0.2× SSC at hybridization temperature. Digoxin was recognized by a sheep anti-DIG directly conjugated with the enzyme alkaline phosphatase (AP) (11093274910, Roche) (1:500) for 60 min at RT. AP converted the applied substrate 4-nitro-blue tetrazolium (NBT)/5-bromo-4-chloro-3′-indolylphosphate (BCIP) (11697471001, Roche) to the soluble substrates NBT and BCIP into a water and alcohol-insoluble dark-blue NBT-BCIP precipitate that appears on slide after 2 h of incubation at 30 °C in the dark. The reaction was stopped, and the slide was counterstained with filtered Nuclear Fast Red. After washing in tap water for 10 min and dehydration with ethanol, the slide was mounted using Eukitt mounting media (03989, Sigma-Aldrich). The ISH result was examined and determined by AIPATHWELL software using H-score to determine the overall percentage of miR-449c-5p positivity across the entire stained tumor sample. The image information was collected using a Pannoramic MIDI system (3DHISTECH).

### Quantification and statistical analysis

Statistical analyses were performed using Prism 8 (GraphPad Software) (www.graphpad.com). All the experiments were repeated three times at least as indicated. Immunoblot analyses were performed with software of IMAGE J. Flow cytometry analysis was performed using the software of FlowJo 7.6.5. Quantitative data are expressed as the mean ± SD. The N, indicated in the figures and figure legends, represent the total number of biological replicates as stated in the figure legends. Two groups of data were compared using two-tailed double sample heteroscedasticity student’s *t* test. *p* < 0.05 was considered statistically significant, and *p*-value was indicated in the figures when statistical analysis was performed.

## Data availability

RNA-Seq raw data generated in this study have been deposited in the GEO database under accession code GSE229941.

## Supporting information

This article contains supporting information.

## Conflict of interest

The authors declare that they have no conflicts of interest with the contents of this article.
