# Supplementary material for: Persistent ER stress causes GPI anchor deficit to convert a GPI-anchored prion protein into pro-PrP via the ATF6–miR449c-5p–PIGV axis
Source: J Biol Chem. 2023 Jun 28;299(8):104982. doi: 10.1016/j.jbc.2023.104982 (PMC10388210; doi:10.1016/j.jbc.2023.104982)
Supplement: Supporting information [file mmc1.pdf]

Supporting information

Persistent ER stress causes GPI anchor deficit to convert a GPI-anchored prion protein (PrP) into pro-PrP via the ATF6-miR449c-5p-PIGV axis

JingFeng Li , SaSa Li, ShuPei Yu, Jie Yang, JingRu Ke, Huan Li, Heng Chen, MingJian Lu, Man-Sun Sy, ZhenXing Gao and Chaoyang Li

Supporting Figures S1-S7

Supporting Tables S1-S7

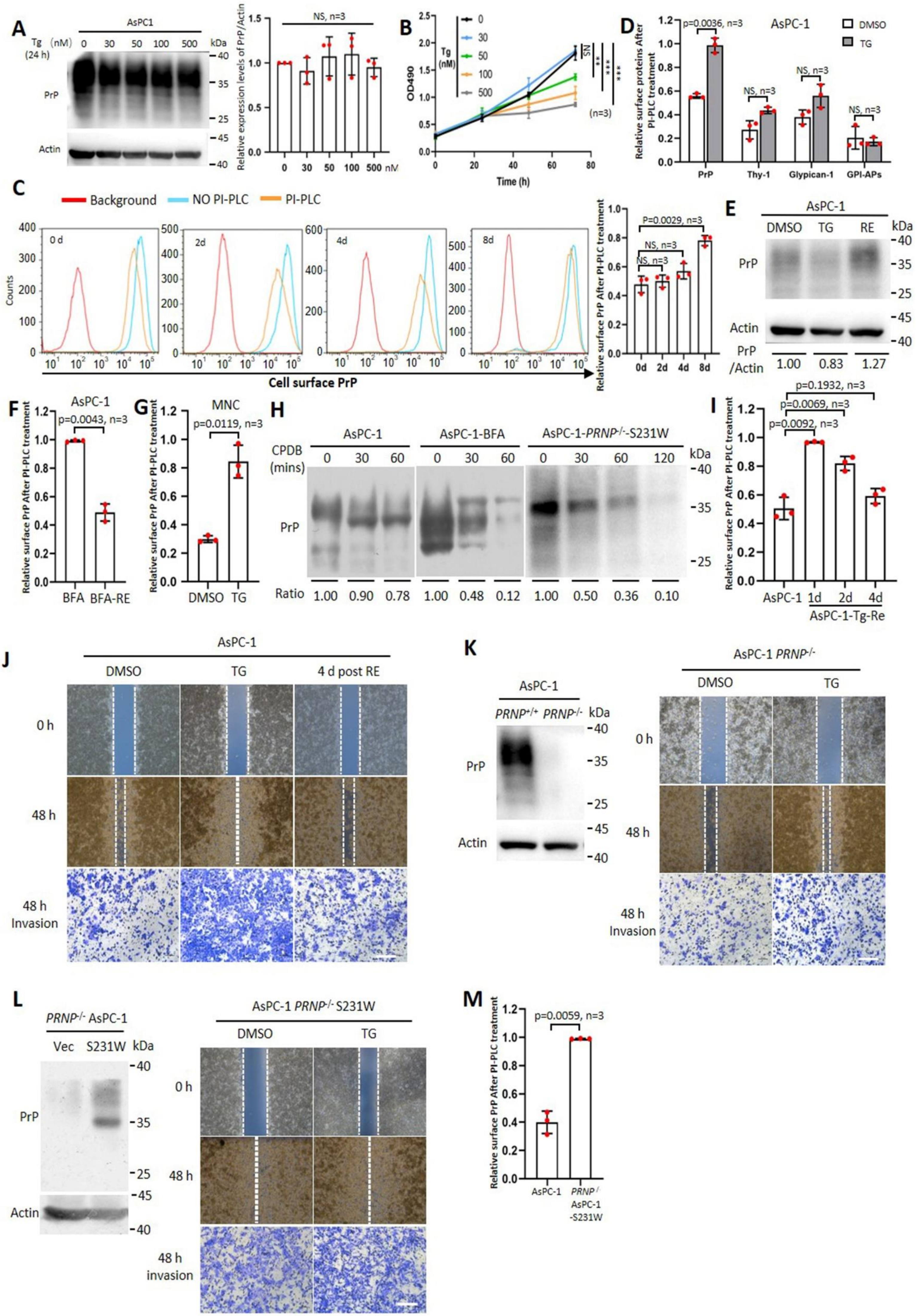

A
AsPC1
Tg (nM) 0 30 50 100 500
(24 h)
PrP
Actin
kDa
Relative expression levels of PrP/Actin
NS, n=3
B
OD490
Tg (nM)
Time (h)
(n=3)
D
p=0.0036, n=3
AsPC-1
DMSO
TG
Relative surface proteins After PI-PLC treatment
PrP
Thy-1
Glypican-1
GPI-APs
C
Background
NO PI-PLC
PI-PLC
Counts
0 d
2d
4d
8d
Cell surface PrP
Relative surface PrP After PI-PLC treatment
p=0.0029, n=3
E
AsPC-1
DMSO TG RE
PrP
Actin
PrP /Actin 1.00 0.83 1.27
F
AsPC-1
p=0.0043, n=3
BFA
BFA-RE
G
MNC
p=0.0119, n=3
DMSO
TG
H
AsPC-1
AsPC-1-BFA
AsPC-1-PRNP-/--S231W
CPDB (mins)
Ratio 1.00 0.90 0.78 1.00 0.48 0.12 1.00 0.50 0.36 0.10
I
p=0.1932, n=3
p=0.0069, n=3
p=0.0092, n=3
AsPC-1 1d 2d 4d
AsPC-1-Tg-Re
J
AsPC-1
DMSO
TG
4 d post RE
0 h
48 h
48 h Invasion
K
AsPC-1
PRNP+/+ PRNP-/-
AsPC-1 PRNP-/-
DMSO
TG
L
PRNP-/- AsPC-1
Vec S231W
AsPC-1 PRNP-/- S231W
48 h invasion
M
p=0.0059, n=3
AsPC-1
PRNP-/- AsPC-1 -S231W

Supporting Figure S1. Related to Figure 1. Persistent Tg and BFA treatment induces pro-PrP accumulation increasing cancer cell migration and invasion in vitro. (A) Immunoblotting analysis of PrP from AsPC-1 cells treated with different concentrations of Tg for 24 hours (left panel). Quantification of immunoblot results (right panel). **(B)** MTS based proliferation assays of AsPC-1 cells cultured with different concentrations of Tg. 30 nM of Tg does not affect the proliferation of AsPC-1 cells. **(C)** Flow cytometry analysis of sensitivity of AsPC-1 cell surface PrP to PI-PLC treatment when cells are treated with 30 nM Tg for different periods as indicated. Statistical analysis of relative surface PrP before and after PI-PLC treatment. Relative surface PrP after PI-PLC treatment is defined as: (geometry mean of PrP intensity after PI-PLC treatment - background geometry mean of PrP intensity) / (geometry mean of PrP intensity before PI-PLC treatment - background geometry mean of PrP intensity). **(D)** Statistical analysis of relative surface PrP or other GPI-APs before and after PI-PLC treatment. Relative surface PrP/Thy-1/Glypican-1 after PI-PLC treatment is defined as: (geometry mean of PrP/Thy-1/Glypican-1 intensity after PI-PLC treatment - background geometry mean of PrP/Thy-1/Glypican-1 intensity) / (geometry mean of PrP/Thy-1/Glypican-1 intensity before PI-PLC treatment - background geometry mean of PrP/Thy-1/Glypican-1 intensity). Relative surface GPI-APs after PI-PLC treatment is defined as: geometry mean of FLARE intensity after PI-PLC treatment/ geometry mean of FLARE intensity before PI-PLC treatment. **(E)** Immunoblotting analysis of PrP from AsPC-1 cells treated with DMSO, Tg for 14 days (TG), or 4 days after removal of Tg from TG (RE). **(F&G)** Statistical analysis of relative surface PrP before and after PI-PLC treatment for AsPC-1 cells treated with DMSO or BFA **(F)** or cell surface PrP from mouse neuron treated with DMSO or Tg **(G)**. **(H)** Immunoblotting analysis of PrP purified by 8B4 from different cell lysates and treated with CPDB

for different periods. **(I)** Statistical analysis of relative surface PrP before and after PI-PLC treatment for TG cells at different days post Tg removal. **(J)** Wound healing and Matrigel pictures showing the migration and invasion of DMSO-treated, or TG, or RE AsPC-1 cells. Scale bars, 200 μm. **(K)** Effects of DMSO- and Tg-treatment (TG) on AsPC-1 cells without *PRNP*. Left: immunoblot analysis of PrP to show silencing of *PRNP* in AsPC-1 cells. Right: wound healing and Matrigel pictures showing the effects of DMSO-and Tg-treatment on the migration and invasion of *PRNP* null AsPC-1 cells. Scale bars, 200 μm. **(L)** Effects of DMSO- and Tg-treatment (TG) on S231W *PRNP* transfected AsPC-1-*PRNP*$^{-/-}$ cells. Left: immunoblot analysis of PrP from cell lysates of vec- or S231W transfected AsPC-1-*PRNP*$^{-/-}$ cells. Right: Wound healing and Matrigel pictures showing the effects of DMSO-and Tg-treatment on the migration and invasion of S231W *PRNP* transfected AsPC-1-*PRNP*$^{-/-}$ cells. Scale bars, 200 μm. Background: Cell not treated with PI-PLC but stained with the same concentration of IgG1 as the primary antibodies indicated. The data were expressed as mean ± SD and were analyzed by two-tailed double sample heteroscedasticity Student's *t* test. NS, not significant.

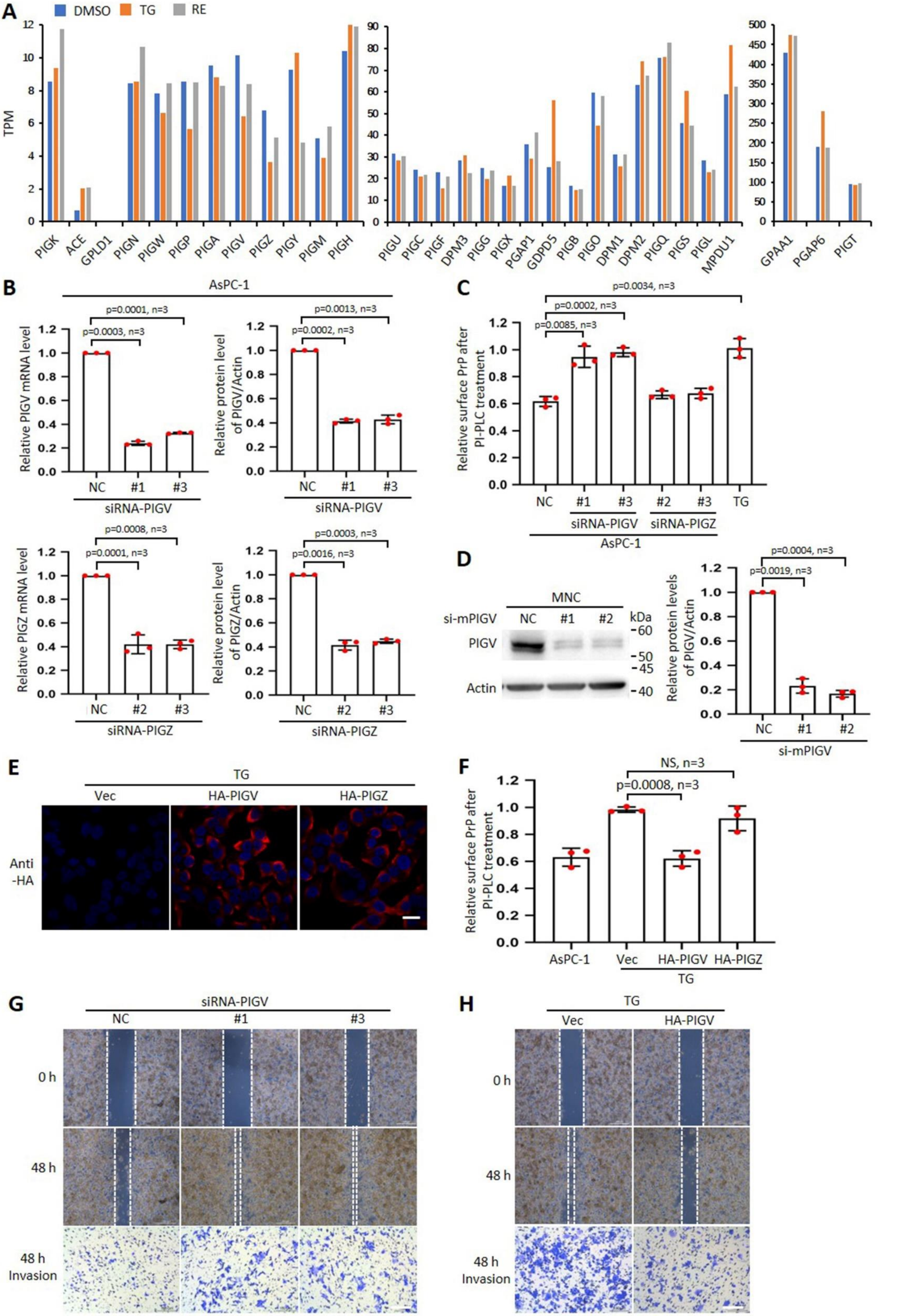
A
DMSO
TG
RE
TPM
PIGK
ACE
GPLD1
PIGN
PIGW
PIGP
PIGA
PIGV
PIGZ
PIGY
PIGM
PIGH
PIGU
PIGC
PIGF
DPM3
PIGG
PIGX
PGAP1
GDPD5
PIGB
PIGO
DPM1
DPM2
PIGQ
PIGS
PIGL
MPDU1
GPAA1
PGAP6
PIGT
B
AsPC-1
p=0.0001, n=3
p=0.0003, n=3
Relative PIGV mRNA level
NC
#1
#3
siRNA-PIGV
p=0.0013, n=3
p=0.0002, n=3
Relative protein level of PIGV/Actin
p=0.0008, n=3
p=0.0001, n=3
Relative PIGZ mRNA level
NC
#2
#3
siRNA-PIGZ
p=0.0003, n=3
p=0.0016, n=3
Relative protein level of PIGZ/Actin
C
p=0.0034, n=3
p=0.0002, n=3
p=0.0085, n=3
Relative surface PrP after PI-PLC treatment
NC
#1
#3
#2
#3
TG
siRNA-PIGV
siRNA-PIGZ
AsPC-1
D
MNC
si-mPIGV
NC
#1
#2
kDa
PIGV
Actin
60
50
45
40
p=0.0004, n=3
p=0.0019, n=3
Relative protein levels of PIGV/Actin
si-mPIGV
E
TG
Vec
HA-PIGV
HA-PIGZ
Anti -HA
F
NS, n=3
p=0.0008, n=3
Relative surface PrP after PI-PLC treatment
AsPC-1
Vec
HA-PIGV
HA-PIGZ
TG
G
siRNA-PIGV
NC
#1
#3
0 h
48 h
48 h Invasion
H
TG
Vec
HA-PIGV
0 h
48 h
48 h Invasion

Supporting Figure S2. Related to Figure 2. Tg treatment reduces PIGV resulting in the accumulation of pro-PrP. (A) Deep-sequencing results of genes related to GPI-anchor synthesis from DMSO treated, - TG or RE cells. **(B)** Statistical analysis of QPCR results of PIGV/PIGZ mRNA levels after silencing PIGV/PIGZ in AsPC-1 cells (Left). Statistical analysis of immunoblot results after silencing PIGV/PIGZ in AsPC-1 cells (Right). **(C)** Statistical analysis of cell surface PrP for AsPC-1 cells silenced PIGV or PIGZ and treated with or without PI-PLC. **(D)** Immunoblot analysis of silencing mouse PIGV in mouse neuronal cells. Statistical analysis of immunoblot results. **(E)** Immunofluorescence staining of HA-tag to reveal the expression of an HA-tagged PIGV (HA-PIGV) or PIGZ (HA-PIGZ) in TG cells. Scale bars, 10 μm. **(F)** Statistical analysis of cell surface PrP for TG cells expressing an HA-PIGV or HA-PIGZ and treated with or without PI-PLC. **(G)** Wound healing and Matrigel analysis of the effects of silencing PIGV in AsPC-1 cells. Scale bars, 200 μm. **(H)** Wound healing and Matrigel pictures showing the effects of expressing an HA-PIGV in TG cells. Scale bars, 200 μm. NC, non-targeting siRNA control. #1 & #3 are two different siRNAs for PIGV while #2 & #3 are two different siRNAs for PIGZ. Vec, empty vector control. The data were expressed as mean ± SD and were analyzed by two-tailed double sample heteroscedasticity Student's *t* test. NS, not significant. Relative cell surface PrP after PI-PLC treatment is defined as in fig. S1C.

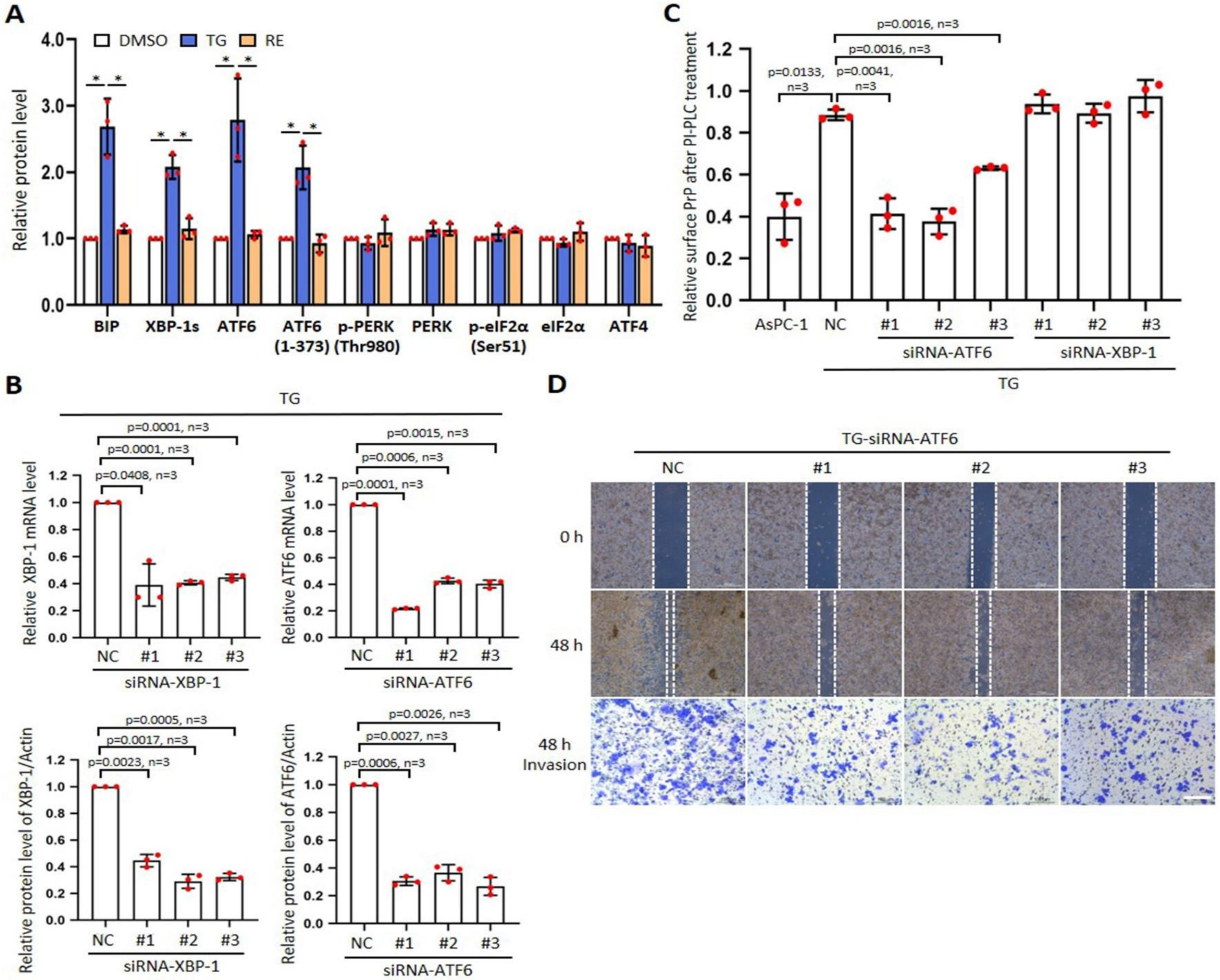
A
DMSO
TG
RE
Relative protein level
BIP
XBP-1s
ATF6
ATF6 (1-373)
p-PERK (Thr980)
PERK
p-eIF2α (Ser51)
eIF2α
ATF4
B
TG
p=0.0001, n=3
p=0.0001, n=3
p=0.0408, n=3
Relative XBP-1 mRNA level
NC #1 #2 #3
siRNA-XBP-1
p=0.0015, n=3
p=0.0006, n=3
p=0.0001, n=3
Relative ATF6 mRNA level
NC #1 #2 #3
siRNA-ATF6
p=0.0005, n=3
p=0.0017, n=3
p=0.0023, n=3
Relative protein level of XBP-1/Actin
NC #1 #2 #3
siRNA-XBP-1
p=0.0026, n=3
p=0.0027, n=3
p=0.0006, n=3
Relative protein level of ATF6/Actin
NC #1 #2 #3
siRNA-ATF6
C
p=0.0016, n=3
p=0.0016, n=3
p=0.0133, n=3
p=0.0041, n=3
Relative surface PrP after PI-PLC treatment
AsPC-1 NC #1 #2 #3 #1 #2 #3
siRNA-ATF6
siRNA-XBP-1
TG
D
TG-siRNA-ATF6
NC #1 #2 #3
0 h
48 h
48 h Invasion

Supporting Figure S3. Related to Figure 3. Tg treatment increases ATF6 to reduce PIGV, resulting in the accumulation of pro-PrP. (A) Statistical analysis of immunoblot results of BiP, XBP-1s, ATF6, c-ATF6, p-PERK, PERK, p-eIF2α, eIF2α, and ATF4 for cell lysates made from DMSO-, TG and RE AsPC-1 cells. **(B)** Statistical analysis of QPCR (upper panels) and immunoblot (bottom panels) results for silencing XBP-1 and ATF6 in TG cells. **(C)** Statistical analysis of cell surface PrP levels in ATF6 and XBP-1 silenced TG cells treated with or without PI-PLC. **(D)** Wound healing and Matrigel analysis of the effects on the migration and invasion of ATF6 silenced TG cells. Scale bars, 200 μm. *$P < 0.05$. NC, non-targeting siRNA control. #1, #2 and #3 are three different siRNAs for XBP-1 or ATF6, respectively. The data were expressed as mean ± SD and were analyzed by two-tailed double sample heteroscedasticity Student's *t* test. Relative cell surface PrP after PI-PLC treatment is defined as in fig. S1C.

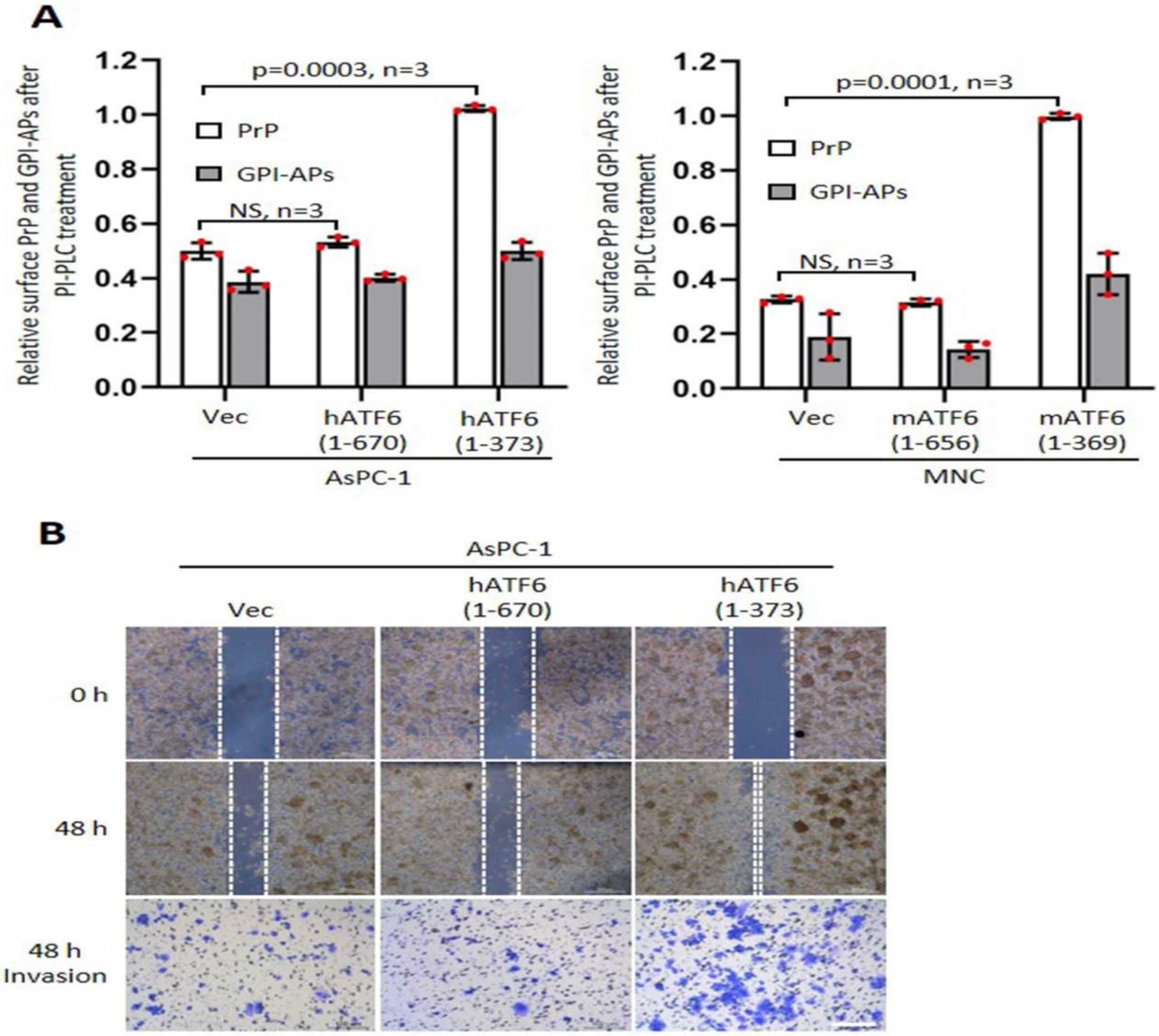

Supporting Figure S4. Related to Figure 4. The accumulation of pro-PrP is induced by Tg via c-ATF6. (A) Statistical analysis of cell surface PrP and GPI-APs after AsPC-1 (left panel) or mouse neuronal (right panel) cells expressing HA-tagged full length (hATF-6 (1-670), mATF6 (1-656)) or c-ATF6 (hATF-6 (1-373), mATF6 (1-369)) and treated with or without PI-PLC. **(B)** Wound healing and Matrigel pictures showing the effects on the migration and invasion of AsPC-1 cells expressing full length or c-ATF6. Scale bars, 200 μm. Vec, empty vector control. The data were expressed as mean ± SD and were analyzed by two-tailed double sample heteroscedasticity Student's *t* test. NS, not significant. Relative cell surface PrP and GPI-APs after PI-PLC treatment is defined as in fig. S1D.

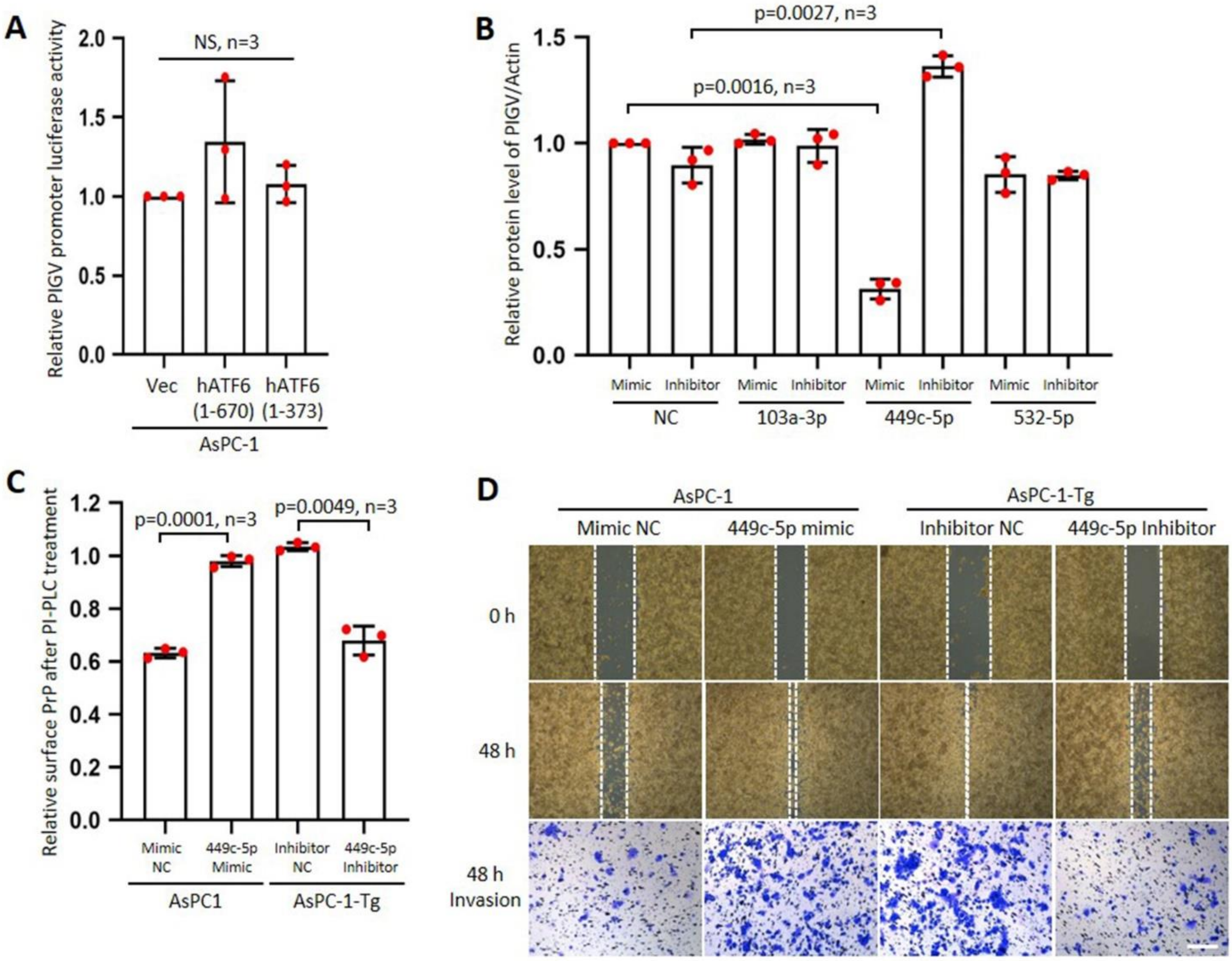

Supporting Figure S5. Related to Figure 5. Hsa-miR-449c-5p suppresses PIGV mRNA level.

(A) Statistical analysis of PIGV promoter luciferase activity in AsPC-1 cells expressing full length or c-ATF6. **(B)** Statistical analysis of PIGV protein levels in AsPC-1 cells treated with mimic or inhibitors of microRNAs 103a-3p, 449c-5p, and 532-5p, respectively. **(C)** Statistical analysis of surface PrP levels of AsPC-1 or AsPC-1-Tg cells when these cells are treated with mimic or inhibitor of microRNA449c-5p and with or without PI-PLC, respectively. **(D)** Wound healing and Matrigel invasion assay pictures showing the effects on the migration and invasion of AsPC-1 or AsPC-1-Tg cells when these cells are treated with mimic or inhibitor of microRNA449c-5p, respectively. Scale bars, 200 μm. NC, non-target miRNA mimic or inhibitor negative control. Vec, empty vector control. The data were expressed as mean ± SD and were analyzed by two-tailed double sample heteroscedasticity Student's *t* test. NS, not significant. Relative cell surface PrP after PI-PLC treatment is defined as in fig. S1C.

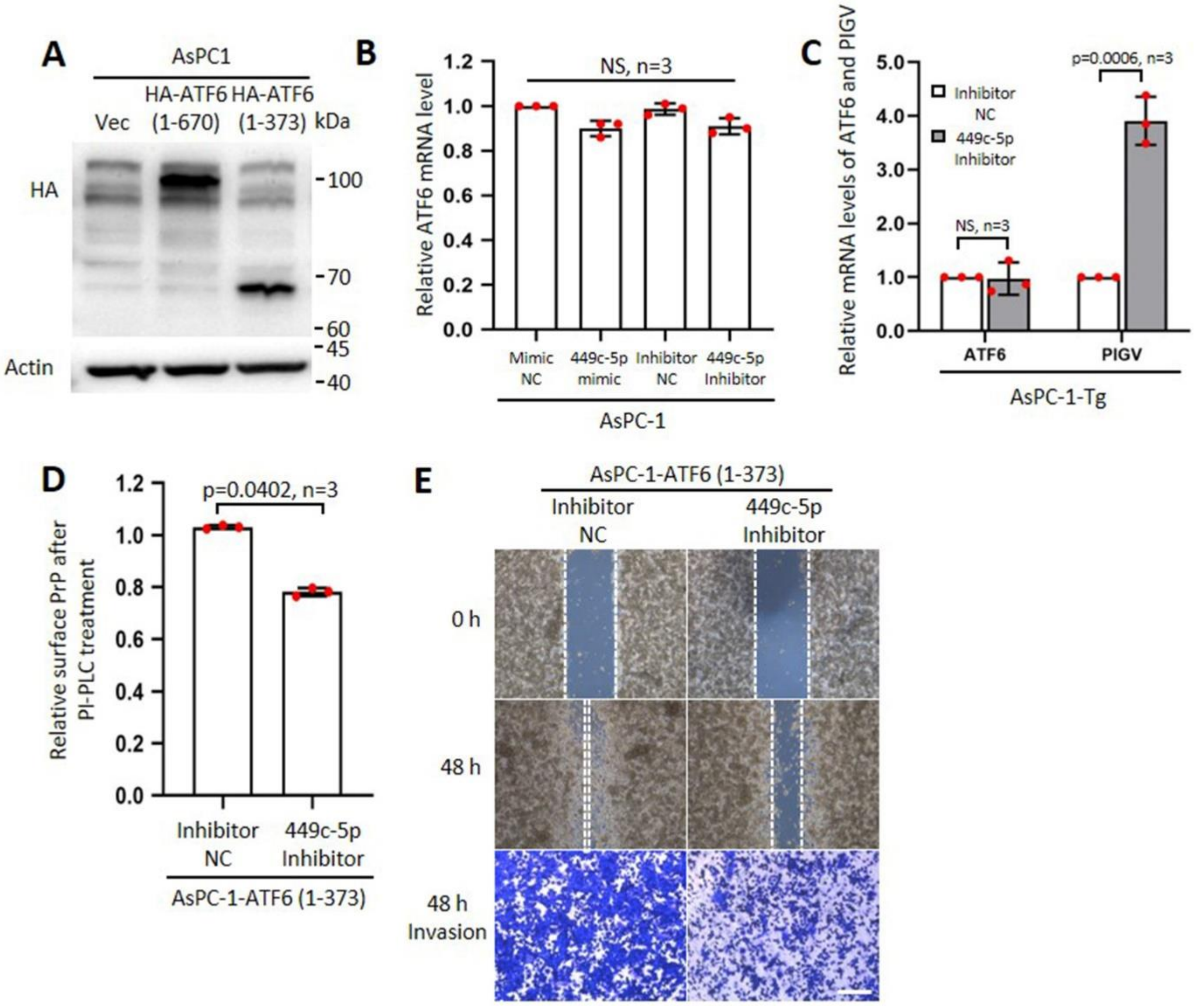

Supporting Figure S6. Related to Figure 6. C-ATF6 reduces PIG V mRNA level via hsa-miR449c-5p. (A) Immunoblot analysis of an HA-tagged full length ATF6 and c-ATF6 in AsPC-1 cells. **(B)** Statistical analysis of QPCR results of ATF6 mRNA levels when AsPC-1 cells are treated with mimic or inhibitor of microRNA449c-5p. **(C)** Statistical analysis of QPCR results of ATF6 and PIGV mRNA levels of AsPC-1 Tg cells when treated with inhibitor of microRNA449c-5p. **(D)** Statistical analysis of cell surface PrP on AsPC-1 cells expressing c-ATF6 and treated with inhibitor of microRNA449c-5p with or without PI-PLC. **(E)** Wound healing and Matrigel pictures showing the effects of expressing c-ATF6 treated with inhibitor of microRNA449c-5p on the migration and invasion of AsPC-1 cells. Scale bars, 200 μm. NC, non-target miRNA mimic or inhibitor control. Vec, empty vector control. The data were expressed as mean ± SD and were analyzed by two-tailed double sample heteroscedasticity Student's *t* test. NS, not significant. Relative cell surface PrP after PI-PLC treatment is defined as in fig. S1C.

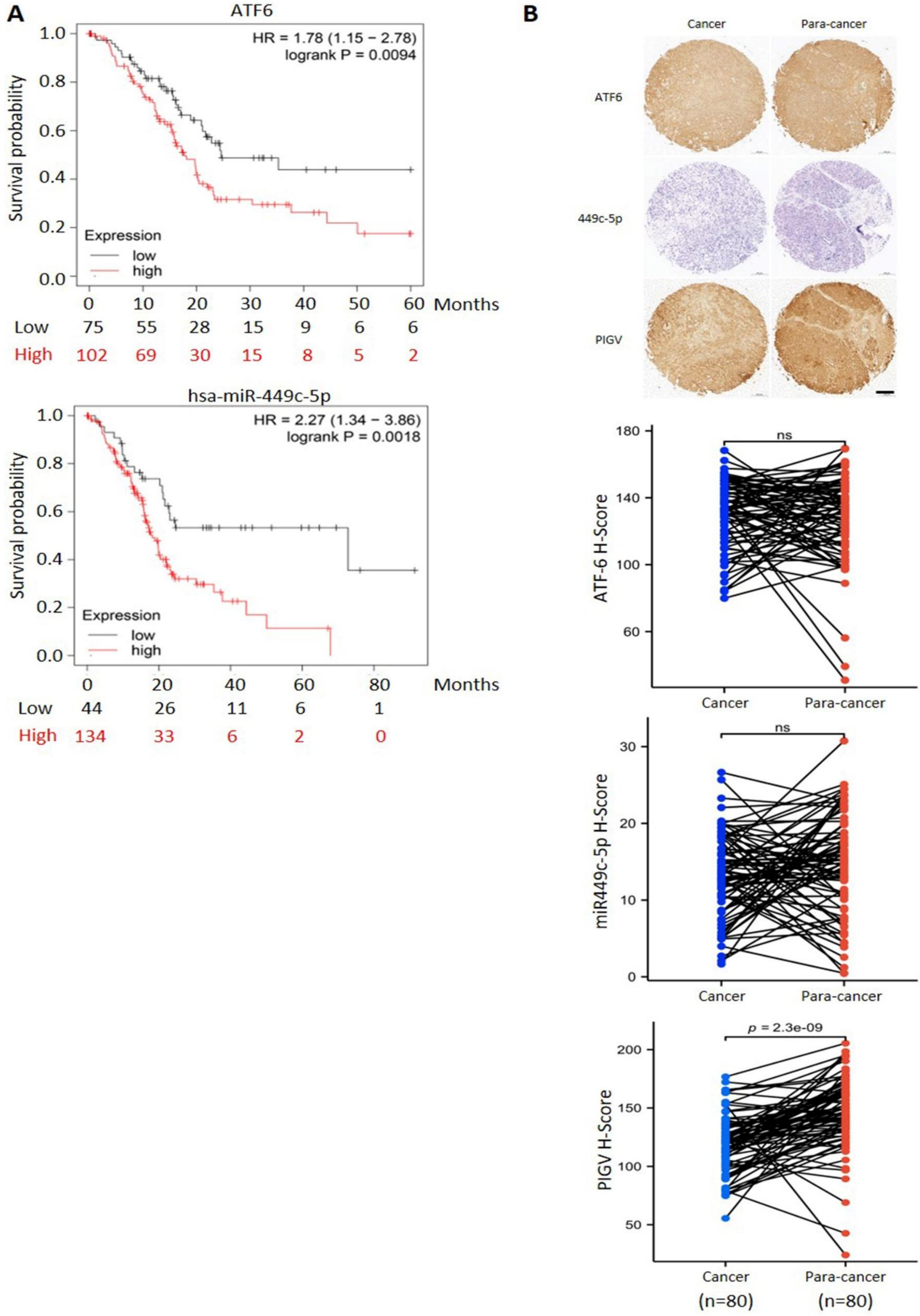
A
ATF6
HR = 1.78 (1.15 − 2.78)
logrank P = 0.0094
Survival probability
Expression
low
high
Months
Low 75 55 28 15 9 6 6
High 102 69 30 15 8 5 2
hsa-miR-449c-5p
HR = 2.27 (1.34 − 3.86)
logrank P = 0.0018
Survival probability
Expression
low
high
Months
Low 44 26 11 6 1
High 134 33 6 2 0
B
Cancer
Para-cancer
ATF6
449c-5p
PIGV
ns
ATF-6 H-Score
Cancer
Para-cancer
ns
miR449c-5p H-Score
Cancer
Para-cancer
p = 2.3e-09
PIGV H-Score
Cancer
Para-cancer
(n=80)
(n=80)

Supporting Figure S7. Related to Figure 7. ATF6-miR449c-5p-PIGV axis contributes to PDAC in humans and marks prognosis in patients with PDAC. (A) Kaplan-Meier survival plot analysis of ATF6 and microRNA449c-5p in PDAC patients. P-value is indicated in each panel and the number of patients with higher or low ATF6 or microRNA449c-5p at specific time points after diagnose is listed. The statistical significance of prognosis analysis was assessed using the Mantel-Cox test. **(B)** Immunohistochemical staining of ATF6 or PIGV, or in situ hybridization of microRNA449c-4p in cancer and paired para-cancer biopsies is shown. Top three panels are matched pictures of stained cancer or para-cancer biopsies. Bottom three panels are statistical analysis of staining results of ATF6, PIGV, and microRNA449c-5p between cancer and paired para-cancer biopsies. Scale bars, 200 μm. The statistical significance of H-Score between cancer and para-cancer was assessed using the Wilcoxon signed rank test. NS, not significant.

Table S1. Related to Figure 5A. List of miRNAs were identified by miRNA sequencing. These miRNAs were significantly up-regulated in AsPC-1-Tg compared to AsPC-1 and were significantly down-regulated in AsPC-1-Tg-Re cells.

miRNAs
hsa-miR-103a-3p, hsa-miR-12136, hsa-miR-125a-5p, hsa-miR-1287-3p, hsa-miR-1306-3p, hsa-miR-135b-3p, hsa-miR-137-3p, hsa-miR-181c-5p, hsa-miR-184, hsa-miR-215-5p, hsa-miR-2278, hsa-miR-3065-3p, hsa-miR-3115, hsa-miR-3187-3p, hsa-miR-3200-3p, hsa-miR-324-5p, hsa-miR-3529-3p, hsa-miR-3617-5p, hsa-miR-362-5p, hsa-miR-365b-3p, hsa-miR-4286, hsa-miR-4466, hsa-miR-4485-3p, hsa-miR-449a, hsa-miR-449c-5p, hsa-miR-450a-5p, hsa-miR-451a, hsa-miR-4638-3p, hsa-miR-4724-5p, hsa-miR-4731-5p, hsa-miR-4775, hsa-miR-500a-5p, hsa-miR-501-3p, hsa-miR-501-5p, hsa-miR-503-5p, hsa-miR-532-5p, hsa-miR-542-5p, hsa-miR-548t-5p, hsa-miR-549a-3p, hsa-miR-549a-5p, hsa-miR-550b-3p, hsa-miR-584-3p, hsa-miR-625-3p, hsa-miR-625-5p, hsa-miR-642a-3p, hsa-miR-6513-3p, hsa-miR-660-5p, hsa-miR-6743-3p, hsa-miR-6824-3p, hsa-miR-6887-3p, hsa-miR-935, hsa-miR-936, hsa-miR-944, hsa-miR-95-3p, novel-hsa-miR11-3p, novel-hsa-miR115-3p, novel-hsa-miR131-3p, novel-hsa-miR214-5p, novel-hsa-miR218-3p, novel-hsa-miR275-3p, novel-hsa-miR275-5p, novel-hsa-miR285-5p, novel-hsa-miR309-5p, novel-hsa-miR97-3p

Table S2. Related to Figure 5A. List of ENCORI predicted miRNAs that target PIGV.

miRNAs
hsa-miR-101-3p, hsa-miR-103a-3p, hsa-miR-107, hsa-miR-34a-5p, hsa-miR-214-3p, hsa-miR-124-3p, hsa-miR-128-3p, hsa-miR-144-3p, hsa-miR-134-5p, hsa-miR-150-5p, hsa-miR-155-5p, hsa-miR-34b-5p, hsa-miR-34c-5p, hsa-miR-365a-3p, hsa-miR-374a-5p, hsa-miR-382-5p, hsa-miR-151a-3p, hsa-miR-331-3p, hsa-miR-449a, hsa-miR-452-5p, hsa-miR-499a-5p, hsa-miR-506-3p, hsa-miR-532-5p, hsa-miR-449b-5p, hsa-miR-653-5p, hsa-miR-770-5p, hsa-miR-28-3p, hsa-miR-214-5p, hsa-miR-532-3p, hsa-miR-874-3p, hsa-miR-374b-5p, hsa-miR-760, hsa-miR-942-5p, hsa-miR-1251-5p, hsa-miR-1269a, hsa-miR-1276, hsa-miR-449c-5p, hsa-miR-2116-3p, hsa-miR-2682-5p, hsa-miR-3118, hsa-miR-3179, hsa-miR-1269b, hsa-miR-4712-5p, hsa-miR-2467-3p

Table S3. Related to Figures S2, 4, S4, and S5. Primers used to construct plasmids.

**Gene**	**Sequence (5' — 3')**
**pHAGE-PIGV-3×HA**	
**Forward**	ATTGCGGCCGCGCCACCATGTGGCCCCAGGACCCATCCCGGA
**Reverse**	ATTGCTAGCTCATGTCCAAGGCAGGAAGTTGC
**pHAGE-PIGZ-3×HA**	
**Forward**	ATTGCGGCCGCGCCACCATGCAGATCTGTGGATCCAGCGTAGCATCTGTAGCAGCT
**Reverse**	ATTGCTAGCTCAGGTTTCTTCCCCCAGCTCCACAATGTGAAGACTGAGGT
**pHAGE-HA-hATF6 (1-670)-3×HA**	

**Forward**	ATTGCGGCCGCGCCACCATGTACCCATACGATGTTCCAGATTACGCTAT GGGGGAGCCGGCTGGGGTTGCCGGCACCAT
**Reverse**	ATTGCTAGCTTGTAATGACTCAGGGATGGTGCTGACAACGTGGGTT
**pHAGE-HA-hATF6 (1-373)-3×HA**	
**Forward**	ATTGCGGCCGCGCCACCATGTACCCATACGATGTTCCAGATTACGCTAT GGGGGAGCCGGCTGGGGTTGCCGGCACCAT
**Reverse**	ATTGCTAGCACTAGGGACTTTAAGCCTCTGGTTCT
**pHAGE-HA-mATF6 (1-656)-3×HA**	
**Forward**	ATTGCGGCCGCGCCACCATGTACCCATACGATGTTCCAGATTACGCTAT GGAGTCGCCTTTTAGTCCGGTTCT
**Reverse**	ATTGCTAGCCTGCAACGACTCAGGGATGGTGC
**pHAGE-HA-mATF6 (1-369)-3×HA**	
**Forward**	ATTGCGGCCGCGCCACCATGTACCCATACGATGTTCCAGATTACGCTAT GGAGTCGCCTTTTAGTCCGGTTCT
**Reverse**	ATTGCTAGCTCTTCGCTTTGGACTTGGGACTTTG
**pGL3-PIGVPRO**	
**Forward**	ATTACGCGTGGGCAAGGATTGTTTCTATCTTGCT
**Reverse**	ATTCTCGAGCCTCCGTTGGTGGATTTCTTGTTTCT

Table S4. Related to Figures 2, S2, 3, S3, 4, 5, and S6. Primers used in mRNA RT-PCR assays.

Gene	Sequence (5’ — 3’)
**PIGA**	
**Forward**	GTTCGGGAGAGAGTCACGATA
**Reverse**	GCTTGTTTGTAAGCACCGAGC
**PIGB**	
**Forward**	TTAGTTCATCAACGAGGTA
**Reverse**	TGGCTGTAATAAGGAGTAG
**PIGC**	
**Forward**	GCCTGAGTTTGAATGAAAGGA
**Reverse**	CCCTCATCCATATACCACCA
**PIGF**	
**Forward**	CATTCCATCACTCTTCTTG
**Reverse**	AGATTGACAGCAGTTACA
**PIGH**	
**Forward**	GAAAGATCCAGTGGAACCACATG
**Reverse**	TTCTGGTGTGCCAGGATCTCCT
**PIGK**	
**Forward**	GCAGCTATAAGGAAGACCAGATG
**Reverse**	GCCCATAATCCCAGAATAAAGCC
**PIGL**	
**Forward**	GCTTCATACGCAGGATGTCCTC
**Reverse**	CGGGAGAAGATAATGTAGAGGCG

**PIGM**	
**Forward**	CGCTTTCCTCTTATACCGCCTG
**Reverse**	GAGGCGACAATAGAGTCCGCAT
**PIGN**	
**Forward**	GAATTGACAGGTTATCTGGA
**Reverse**	GGAGAATGCCTTCCTATG
**PIGO**	
**Forward**	GTATTCAGATTCTGGCCTGTG
**Reverse**	CTGTCTTGCAGATCATCCAG
**PIGP**	
**Forward**	TACCTCCTTATTGCTATAGT
**Reverse**	TAGTTATCTGTGATTGTATGG
**PIGQ**	
**Forward**	CTGTGGATCAGCTACATCCA
**Reverse**	CCAGGTCATAGGAACAGGAG
**PIGS**	
**Forward**	GCGGCTACACACCTAGAGG
**Reverse**	CTGGGAGTAAGGCAACGAGG
**PIGT**	
**Forward**	AGCGGTACGTGAGTGGCTAT
**Reverse**	GATACCAGGGTACGGTGTCCA
**PIGU**	
**Forward**	TCTGGCCGAGTTCATTTCCG
**Reverse**	CCAAGTCCAACAGTGAAAGGC
**PIGV**	
**Forward**	CATGTTCAGGTTCTCACCAG
**Reverse**	GCCTAGAATGTATCGTGTGAC
**PIGW**	
**Forward**	CACCATTTGGAGTACGTGAG
**Reverse**	AGGAGGATAAATGAAGCCCA
**PIGX**	
**Forward**	CATAACAGAGGCAGTGATGG
**Reverse**	AATGCACTGTGAATCTCGTC
**PIGY**	
**Forward**	GCTCTGACTTATAACTGCT
**Reverse**	ACTCTTAGATGAATGGAATAAGA
**DPM1**	
**Forward**	ACAGAATTCTTCTAAGACCACG
**Reverse**	CTCCATTTCCTTTGTAGCGA
**DPM2**	
**Forward**	TTAGCCTGATCATCTTCACCT
**Reverse**	ATGAACAGTCCCACAAACAG
**DPM3**	

**Forward**	ATGACGAAATTAGCGCAGTGG
**Reverse**	GCGGACACCAGCAAGTAGG
**GPAA1**	
**Forward**	CCGGGTGGTAAGCACACAG
**Reverse**	GGGCAGCATAAAGGGTCCG
**MPDU1**	
**Forward**	TCACTTCTAGTAAAGCTGCCC
**Reverse**	TCTGGAGCATCAGGAATAAGG
**PGAP1**	
**Forward**	TTCCTGCTTATGTCGTATC
**Reverse**	GTATGGTTTGGCTTCTTT
**PIGG**	
**Forward**	ATGCCCTACACAACTTACCTTG
**Reverse**	CTCCCCGTCATCAATGCCTT
**PIGZ**	
**Forward**	CTGTCTGGTTCCTACGTCACC
**Reverse**	CCCACGTTACATGGGAGGATA
**GPLD1**	
**Forward**	CTAGAACACCAGGATGCGTATC
**Reverse**	GGAGTCCAGTGAGTGCTCTC
**ACE**	
**Forward**	GGAGGAATATGACCGGACATCC
**Reverse**	TGGTTGGCTATTTGCATGTTCTT
**GDPD5**	
**Forward**	GTACCTCTACAACCGCATGG
**Reverse**	AATGTGACATAGTGCCAGGAC
**PGAP6**	
**Forward**	AGAAGATCGAGTTGAAGGGCT
**Reverse**	CGCGTGTAATCGGGGACAA
**XBP-1**	
**Forward**	ATGGATTCTGGCGGTATTGACT
**Reverse**	GGAGGCTGGTAAGGAACTGG
**ATF6**	
**Forward**	TCCTCGGTCAGTGGACTCTTA
**Reverse**	CTTGGGCTGAATTGAAGGTTTTG
**PRNP**	
**Forward**	GTGACTATGAGGACCGTTACTATC
**Reverse**	TGACCGTGTGCTGCTTGA
**hActin**	
**Forward**	ATCGTGCGTGACATTAAGGAG
**Reverse**	GGAAGGAAGGCTGGAAGAGT
**mPIGV**	

**Forward**	TGGCTCCCTTGGGAATTTAGA
**Reverse**	GCGTGGTGATCTGGGATGATG
**mActin**	
**Forward**	CCGCCCTAGACATCAGGGT
**Reverse**	TCTTCTGGTGCTACTCGAAGC

Table S5. Related to Figures 2, S2, 3, and S3. siRNA sequence.

Gene	Sequence (5' — 3')
**PIGV**	
**NC**	UUCUCCGAACGUGUCACGU
**#1**	CCUGAGCCUUUGGUACAGUUA
**#3**	CCGCUGUUGAGAUCCUUAATT
**PIGZ**	
**NC**	UUCUCCGAACGUGUCACGU
**#2**	CUGUCAACUUCUUGCACUATT
**#3**	GGUCCUGCUUUGUAGUCCATT
**ATF6**	
**NC**	UUCUCCGAACGUGUCACGU
**#1**	GUGAGCUACAAGUGUAUUATT
**#2**	GGAGACAGCAACGUAUGAUTT
**#3**	GUGGACUCUUAUUCUUCAATT
**XBP-1**	
**NC**	UUCUCCGAACGUGUCACGU
**#1**	CACCCUGAAUUCAUUGUCUTT
**#2**	GCGGUAUUGACUCUUCAGATT
**#3**	GUUGAGAACCAGGAGUUAATT
**mPIGV**	
**#1**	GAUGCGUUCUGGUCUACUU
**#2**	CAUCCUGACUCUAGUGCUA

NC: non-targeting siRNA.

Table S6. Related to Figures 5 and 6. Primer sets used in miRNA RT-PCR assay.

miRNA	Product name	Catalogue code
**has-miR-103a-3p**	Bulge-Loop has-miR-103a-3p qPCR Primer Set	MQPS0000423-1
**has-miR-449a**	Bulge-Loop has-miR-449a qPCR Primer Set	MQPS0001416-1
**has-miR-449c-5p**	Bulge-Loop has-miR-449c-5p qPCR Primer Set	MQPS0001420-1
**has-miR-532-5p**	Bulge-Loop has-miR-532-5p qPCR Primer Set	MQPS0001792-1
**U6**	Bulge-Loop U6 qPCR Primer Set	MQPS0000002-1

Table S7. Related to Figures 5, S5, 6, and S6. miRNA mimic and inhibitor.

miRNA	Product name	Catalogue code
**has-miR-103a-3p**		

**mimic**	micrON has-miR-103a-3p mimic	miR10000101
**inhibitor**	micrOFF has-miR-103a-3p inhibitor	miR20000101
**has-miR-449c-5p**		
**mimic**	micrON has-miR-449c-5p mimic	miR10010251
**inhibitor**	micrOFF has-miR-449c-5p inhibitor	miR20010251
**has-miR-532-5p**		
**mimic**	micrON has-miR-532-5p mimic	miR10002888
**inhibitor**	micrOFF has-miR-532-5p inhibitor	miR20002888
**NC**		
**mimic**	micrON NC mimic	miR1N0000001-1
**inhibitor**	micrOFF NC inhibitor	MiR2N0000001-1

NC: non-targeting miRNA.
